# Seasonal dynamics of cell-to-cell transport in angiosperm wood

**DOI:** 10.1093/jxb/erad469

**Published:** 2023-11-23

**Authors:** Aleksandra Słupianek, Elżbieta Myśkow, Anna Kasprowicz-Maluśki, Alicja Dolzblasz, Roma Żytkowiak, Magdalena Turzańska, Katarzyna Sokołowska

**Affiliations:** Department of Plant Developmental Biology, Faculty of Biological Sciences, University of Wrocław, Kanonia 6/8, 50-328 Wrocław, Poland; Department of Plant Developmental Biology, Faculty of Biological Sciences, University of Wrocław, Kanonia 6/8, 50-328 Wrocław, Poland; Department of Molecular and Cellular Biology, Institute of Molecular Biology and Biotechnology, Faculty of Biology, Adam Mickiewicz University, Uniwersytetu Poznańskiego 6, Poznań 61-614, Poland; Department of Plant Developmental Biology, Faculty of Biological Sciences, University of Wrocław, Kanonia 6/8, 50-328 Wrocław, Poland; Institute of Dendrology, Polish Academy of Sciences, Parkowa 5, 62-035 Kórnik, Poland; Department of Plant Developmental Biology, Faculty of Biological Sciences, University of Wrocław, Kanonia 6/8, 50-328 Wrocław, Poland; Department of Plant Developmental Biology, Faculty of Biological Sciences, University of Wrocław, Kanonia 6/8, 50-328 Wrocław, Poland; Cardiff University, UK

**Keywords:** *Acer pseudoplatanus*, endocytosis, *Fraxinus excelsior*, plasmodesmata, *Populus tremula* × *tremuloides*, vessel-associated cells, wood, xylem parenchyma cells

## Abstract

This study describes the seasonal changes in cell-to-cell transport in three selected angiosperm tree species, *Acer pseudoplatanus* (maple), *Fraxinus excelsior* (ash), and *Populus tremula* × *tremuloides* (poplar), with an emphasis on the living wood component, xylem parenchyma cells (XPCs). We performed anatomical studies, dye loading through the vascular system, measurements of non-structural carbohydrate content, immunocytochemistry, inhibitory assays and quantitative real-time PCR to analyse the transport mechanisms and seasonal variations in wood. The abundance of membrane dye in wood varied seasonally along with seasonally changing tree phenology, cambial activity, and non-structural carbohydrate content. Moreover, dyes internalized in vessel-associated cells and ‘trapped’ in the endomembrane system are transported farther between other XPCs via plasmodesmata. Finally, various transport mechanisms based on clathrin-mediated and clathrin-independent endocytosis, and membrane transporters, operate in wood, and their involvement is species and/or season dependent. Our study highlights the importance of XPCs in seasonally changing cell-to-cell transport in both ring-porous (ash) and diffuse-porous (maple, poplar) tree species, and demonstrates the involvement of both endocytosis and plasmodesmata in intercellular communication in angiosperm wood.

## Teaser

In ring- and diffuse-porous wood, plasmodesmata are involved in intercellular transport of large-molecular-mass dyes internalized from vessels to vessel-associated cells; endocytic membrane dye abundance and the contribution of different transport mechanisms are season dependent.

## Introduction

Short- and long-distance communication is challenging for trees and other perennial organisms with exceptional height and enhanced lateral growth. To transport molecules in axial and radial directions, trees use conductive tissues, among which the secondary xylem (wood) is particularly important (Holbrook and Zwieniecki, 2011; Carlquist, 2012). Within a complex wood structure, transport proceeds along and between the apoplasmic and symplasmic routes. The apoplasm forms a space beyond the plasmalemma consisting of cell walls, gas- and water-filled intercellular spaces, and the lumen of tracheary elements (e.g. vessel elements). The symplasm consists of protoplasts connected by plasmodesmata (Canny, 1993; Evert, 2006; Moreau *et al*., 2021) and, in wood, consists of living xylem parenchyma cells (XPCs) (Frey-Wyssling and Bosshard, 1959; Kasuga *et al.*, 2008). Consequently, transport studies in wood require interdisciplinary approaches that often include anatomical studies linked to the identification and quantification of structural and non-structural compounds, dye loading, and molecular methods (Choat *et al*., 2007; Plavcová *et al*., 2013; Pfautsch *et al.*, 2015; Hartmann and Trumbore, 2016; Blokhina *et al.*, 2019; Li *et al.*, 2021b).

The xylem parenchyma is a crucial component of wood involved in the cell-to-cell transport of water, ions, sugars, amino acids, and proteins (Zwieniecki *et al*., 2004; Couturier *et al*., 2010; Fuchs *et al*., 2010; Sokołowska, 2013; Carlquist, 2018; Mahboubi and Niittylä, 2018; van Bel, 2021; Zhang *et al*., 2021), acting as storage sites (Hartmann and Trumbore, 2016; Pratt and Jacobsen 2017), forming anatomical and defence barriers during the responses against decay and fungi (Morris *et al*., 2020), and modulating xylem sap flow (Nardini *et al*., 2011; Secchi *et al*., 2011; Pagliarani *et al*., 2019). Moreover, XPC activity varies annually (Sauter *et al*., 1996; Ache *et al*., 2009; Larisch *et al*., 2012; Furze *et al*., 2019), thus serving as a good example of seasonal changes in tree activity (Ziegler, 1964; Sauter and van Cleve, 1994; Hoch *et al.*, 2003; Ray and Savage, 2021; van Bel, 2021). Therefore, a comprehensive understanding of transport mechanisms in trees requires consideration of seasonal activity, species, wood type, and habit-related variances (Sauter and van Cleve, 1994; Sass-Klaassen *et al*., 2011; Prislan *et al*., 2013; Furze *et al*., 2020; Balzano *et al*., 2021; Chen *et al*., 2022).

Two main wood types can be found in angiosperm trees: ring-porous type, characterized by the presence of large earlywood vessels and smaller latewood elements, and diffuse-porous type, with vessels of similar diameters uniformly distributed within the annual growth ring (Wheeler *et al*., 1989; Schweingruber and Baas, 2011). In addition, two functional types of living XPCs, belonging to a three-dimensional system of axial and ray parenchyma, have been identified in angiosperm wood. The first type, vessel-associated cells (VACs), abut vessel elements and make direct contact with them via specialized contact pits. The second type of parenchyma cells, non-vessel-associated cells (nonVACs), are distant from the vessels and cannot directly exchange molecules with them (Czaninski, 1977; Murakami *et al*., 1999; Morris *et al*., 2018). Recently, we discovered that the internalization of dyes, even those with a high molecular mass, from vessels to neighbouring VACs occurs via clathrin-mediated endocytosis (CME) as well as clathrin-independent pathways (Słupianek *et al*., 2019). However, the fate of the internalized dyes remains unclear. It is generally known that the xylem parenchyma is interconnected via numerous plasmodesmata located in simple pits (Sauter and Kloth, 1986; Chaffey and Barlow, 2001; Barnett, 2006) and plays a key role in symplasmic transport both within wood (Sokołowska and Zagórska-Marek 2012; Kuroda *et al*., 2018) and between wood and outwardly located tissues, such as phloem and bark (Mahboubi *et al*., 2013; Pfautsch *et al*., 2015; Treydte *et al.*, 2021; van Bel, 2021). Therefore, we can assume that molecules that were transported from vessels into the VACs and ‘trapped’ in the endomembrane system can move farther using plasmodesmata that connect VACs to other XPCs. This is similar to data obtained for trichomes and epidermal cells, where intercellular movement was revealed with the use of fluorescent dyes injected into the endomembrane system (Cantrill *et al.*, 1999) or specifically into the lumina of endoplasmic reticulum (Barton *et al*., 2011). To the best of our knowledge, cell-to-cell transport within the endomembrane system of angiosperm wood has not yet been experimentally validated.

In the present study, we aimed to determine whether, in the wood of ring-porous (ash) and diffuse-porous (maple and poplar) tree species, dyes internalized into the endomembrane system (during their transport from vessel elements into VACs) can subsequently move from VACs to nonVACs, and to elucidate the possible mechanism of this transport. Moreover, we wanted to determine whether these two stages of dye transport (from vessel elements to VACs, and from VACs to nonVACs) change seasonally, and whether wood type- and season-related dependencies impact various transport mechanisms operating in angiosperm wood.

## Materials and methods

### Plant material

Saplings of *Acer pseudoplatanus* L. (maple, *n*=64) and *Fraxinus excelsior* L. (ash, *n*=80) with roots, and branches of *Populus tremula* L. × *tremuloides* Michx (poplar, *n*=92) were harvested from natural habitats in Wrocław (Poland) and used for the experiments on the same day. The plants and branches had secondary xylem characterized by at least two annual rings.

Based on tree phenology and diel air temperature, four seasons were differentiated in this study: winter (<5 °C; lack of leaves), spring (5–15 °C; bud opening and leaf unfolding), summer (>15 °C, fully developed leaves), and autumn (<10 °C; leaves falling) (Supplementary Fig. S1). Maple and ash trees used for anatomical studies, analyses of non-structural carbohydrate (NSC) content, and membrane dye abundance in wood were collected in 2016, from 18 January [the 18th day of the year (18 DOY)] until 6 December (341 DOY), at 2-week intervals (24 time points in total). At each of the 24 time points, two maple and two ash trees were analysed. Soluble sugars and sugar alcohols were analysed in ash (*n*=15) and maple (*n*=15) stems collected in 2016; inhibitory assays were performed on ash (*n*=16) and maple (*n*=16) trees harvested during 2017–2019 and on poplar branches (*n*=24) during 2018–2020 (poplar branches were also used for anatomical studies); and gene expression analyses of poplar (*n*=34) branches collected during 2018–2019 were all performed in spring, summer, autumn, and winter. Additional loading experiments with membrane dyes or Texas Red 3 kDa (3TR) were performed on ash (*n*=13) and poplar (*n*=13) plant materials harvested during the spring and summer of 2017–2019. Fluorescence recovery after photobleaching (FRAP) experiments were conducted using ash (*n*=3) and poplar (*n*=4) trees harvested during the spring and summer of 2019.

### Anatomical studies

Small fragments (0.5 × 0.5 × 0.2 cm) of ash (*n*=48), maple (*n*=48), and poplar (*n*=5) stem internodes were embedded in paraffin (O’Brien and McCully, 1981). Transverse and longitudinal sections (8 µm thick) were prepared using a rotary microtome (Leica RM 2135, Germany) and stained with Alcian Blue-Safranin O solution, mounted in Euparal, and observed under a bright-field microscope with a polarization adapter. The cambial activity and processes of wood formation in ash and maple were analysed according to Myśkow *et al*. (2021). Cambial cells were characterized by a rectangular shape and thin cell walls. The border between the cambial and wood regions was easy to distinguish in the dormant cambium owing to the presence of the thick cell walls of fully differentiated wood cells. The onset of cambial divisions was indicated by the presence of thin periclinal walls in the cambial cells. In contrast, the lack of these divisions confirmed the end of cambial activity. During the vegetative season, when new wood cells were formed, different stages of xylem cell differentiation (e.g. post-cambial growth, secondary cell wall formation, lignification, and maturation) were determined. The cells entering the post-cambial growth stage were characterized by the presence of thin cell walls and increased radial dimension compared with cambial initials. The cells that began to form the secondary cell wall were discerned under polarized light. Lignification was revealed by the red colour of the cell wall obtained by safranin staining, whereas the appearance of contentless vessel elements and fibres distinguished their maturity.

### Measurements of non-structural carbohydrates and metabolite content analysis

Ash (*n*=48) and maple (*n*=48) wood samples were isolated from the middle stem internodes and the inner pith, cambium, and bark were carefully removed. For each time point analysed at 2-week intervals, wood samples with a minimum of 0.05 g dry mass were taken from two individual trees of both ash and maple, oven-dried (60–65 °C for 24 h/48 h), milled, and used to determine the concentration (mg g^–1^ dry weight) of NSCs (starch and soluble sugars) using a previously described colorimetric method (Oleksyn *et al*., 2000).

To identify particular soluble sugars and sugar alcohols in ash and maple wood, and to determine possible seasonal changes in their content for each of the five time points, that is, winter (46 DOY and 341 DOY), spring (130 DOY), summer (201 DOY), and autumn (284 DOY), additional samples of isolated wood (*n*=6 for ash and *n*=6 for maple) were collected, frozen (–80 °C), ground, subjected to methanol-based extraction, and analysed using gas chromatography-mass spectrometry (GC-MS) according to Yoon *et al.* (2020). Frozen and powdered wood samples (130 mg) were flooded with 1.75 ml of 80% cooled methanol supplemented with 25 µm ribitol (1 mg ml^–1^), vortexed at 950 rpm for 10 min at room temperature (RT) followed by 10 min of centrifugation at 11 000 *g* at 4 °C, and then dried in a vacuum concentrator at RT. Each dried sample was supplemented with 50 µl methoxyamine (20 mg ml^–1^ in dry pyridine) and incubated in a thermomixer for 1.5 hours at 37 °C. After 1 min of centrifugation at 11 000 *g*, 80 µl of *N*-methyl-*N*-(trimethylsilyl)trifluoroacetamide (MSTFA, Sigma-Aldrich) reagent was added to each sample, and samples were incubated once more for 30 min at 37 °C before being centrifuged at 11 000 g for 10 min and then transferred to conical glass vials. A gas chromatograph coupled to a mass spectrometer (TRACE 1310 GC oven with TSQ8000 triplequad MS, Thermo Scientific, USA) with a DB-5MS column (30 m × 0.25 mm, d_f_ 0.25 µm, J&W Scientific, Agilent Technologies, Palo Alto, CA, USA) was used to determine the levels of endogenous carbohydrates. A temperature gradient was applied in the following manner to separate volatile compounds: 2 min at 70 °C, then an increase of 10 °C min^–1^ up to 300 °C, then 10 min at 300 °C. For sample injection, a programmable temperature vaporizing injector with a temperature range of 60–250 °C was used. The source and transfer line temperatures were both set to 250 °C. Spectra were acquired at an electron energy of 70 eV, in the m/z range of 50–850 in EI+ mode. The retention index mixture containing alkanes was run prior to relevant analyses. Thermo Xcalibur 2.2 software was used for instrument control, preparation, and data acquisition. Automatic peak detection, mass spectrum deconvolution, retention index calculation and library searches were performed using MSDial 4.9. Retention indexes for each compound were calculated based on the alkane series mixture (C-10 to C-36) analysis. Metabolites were identified manually using the NIST library and by library searches using a dedicated database from RIKEN (MSDial) containing 28 220 records. The analyte was considered identified when the quality threshold was passed, that is, at a similarity index >700 and a matching retention index ±40. Artefacts (alkanes, column bleed, plasticizers, MSTFA, and reagents) were identified analogously and then excluded from further analyses. The obtained data were normalized against the sum of the chromatographic peak areas (using the Total Ion Chromatogram approach), and the resulting tables were transferred into Perseus 1.6.15 software (Max Planck Institute of Biochemistry, Germany). The ion intensities were log transformed and filtered for blanks in samples. The missing values in the Perseus data table were replaced (by imputation with values calculated from a normal distribution), and matrices prepared in this way were used for the statistical calculations. Values were *Z*-scored from each row in heatmaps generated by using Heatmapper software according to Babicki *et al.* (2016).

### Dye loading studies and inhibitory assays

Aqueous working solutions of fluorescent dyes, membrane-impermeable 3 kDa dextran 3TR (Invitrogen), and two fixable analogues of lipophilic styryl membrane dyes, FM 4-64FX and FM 1-43FX (Invitrogen), were used at concentrations of 0.01% (3TR) and 50 µM (FM 4-64FX and FM 1-43FX) in all experiments (Słupianek *et al*., 2019), except for FRAP with ash, where 25 µM FM 4-64FX was used. Tyrphostin A23 (TA23) (Sigma-Aldrich), an inhibitor of CME, was used at 50 µM (Hao *et al*., 2014).

The dye loading and inhibitor treatments were performed as described by Słupianek *et al*. (2019). Briefly, before the loading procedure, the maple stems, ash stems, and poplar branches were cut transversely under water and left in tap water for approximately 30 min. The surfaces of transversely cut stems and branches were either (i) immersed directly in working solutions of the analysed dyes (FM 4-64FX, FM 1-43FX, or 3TR) for 1 h, alternating with tap water every 10 min (in control plants only tap water was applied), or (ii) immersed in the TA23 inhibitor for 2 h and then in a mixture of TA23 and FM 4-64FX dye for 1 h, alternating with TA23 solution alone (in control plants tap water was used instead of TA23). For all loading studies with the 3TR and FM dyes, basal fragments of the stem were cut prior to dye application and used to estimate tissue autofluorescence (blank controls). After completion of the loading procedure, the basal fragments of stems and branches were cut off, fixed in 4% paraformaldehyde (Affymetrix) in phosphate-buffered saline (PBS) for 48 h at 4 °C, and then stored in PBS at 4 °C for further use. Dye abundance in wood and its changes upon treatment with the inhibitor were quantified by calculating the number of VACs and nonVACs with the signal of internalized dye per analysed wood area using transverse sections (35 µm thick) of stems and branches prepared from fixed fragments using a vibratome (Leica VT 1200S). All sections, except for the first three, were successively collected from six different stem locations obtained from a distance of 100–1000 µm from the site of dye application, then placed in an appropriate order on microscope slides with a drop of a mixture of PBS buffer and glycerol (1:1) and analysed under an epifluorescence microscope (see Microscopy, below). Measurements were performed in at least two randomly selected wood areas (extending from the pith to the cambium along the radial rows) prepared from at least two stem locations. Images were stitched together in Helicon Focus 5.3 (Helicon Soft, Ukraine), and overlapped in Corel Paint Shop Pro (Corel, Canada) to obtain larger wood maps from the pith to the cambium along xylem rays. The analysed wood areas had an average size of at least 0.4 mm^2^.

### Ultrastructural and immunogold studies

The immunogold assay was performed as described by Bilska-Kos *et al*. (2017, 2020). After 3TR loading, small (0.1 × 0.1 × 0.05 cm) basal parts of ash stems (*n*=3) and poplar branches (*n*=3) were fixed in a mixture of 5% glutaraldehyde (Sigma-Aldrich) and 4% methanol-free formaldehyde (Thermo Fisher Scientific, Waltham, MA, USA) in 25 mM phosphate buffer, first under low vacuum for 2 h at RT, then overnight at 4 °C. The material was dehydrated through a graded ethanol series (10–100%), gradually embedded (10%, 25%, 50%, 75%, and 100%) in LR White resin (TAAB Laboratories Equipment Ltd) for 7 d, and polymerized in 100% LR White for 48 h at 60 °C. Ultrathin sections were prepared using an ultramicrotome (Reichert Ultracut E, Munich, Germany) and mounted on nickel grids. Immunogold localization for TR was performed using a rabbit polyclonal anti-TR antibody (Thermo Fisher Scientific) and a secondary antibody conjugated to 10 nm gold particles (anti-rabbit, Thermo Fisher Scientific). In the first stage, blocking was performed using a 4% BSA solution (Aurion Immuno Gold Reagents & Accessories, Wageningen, The Netherlands) in PBS buffer for 1 h at RT. After this, samples were incubated overnight at RT with primary antibodies (diluted 1:200 in PBS buffer with 2% BSA). After a series of wash cycles, the material was incubated for 2 h at RT with a secondary antibody at 1:100 dilution in PBS. Finally, samples were contrasted with 2% uranyl acetate for 20 min and analysed by transmission electron microscopy (TEM). To localize callose (1,3-β-d-glucan), an immunofluorescence assay was performed on transverse vibratome stem sections prepared from trees that were first loaded with the membrane dye FM 4-64FX according to the procedure described above (see Dye loading studies and inhibitory assays). Subsequently, stem fragments were fixed in 4% PFA for 48 h at 4 °C, as described above. To increase the permeability of antibodies (through thick secondary cell walls), the sections were first incubated in an aqueous solution of 1% macerozyme for 30 min at 37 °C. Blocking was performed in 2% skimmed milk (SM) (Fluka Chemie GmbH, Buchs, Switzerland) in PBS buffer for 1 h at RT. Primary monoclonal antibody against 1,3-β-d-glucan (Biosupplies, Australia, Melbourne, Australia) was diluted 1:100 in PBS with 1% SM and incubated with the sections overnight at RT with gentle shaking. Subsequently, sections were washed and incubated for 2 h at RT with 1:50 diluted goat anti-mouse 405 (Thermo Fisher Scientific) in PBS with 1% SM. After incubation, the sections were washed first in PBS with 1% SM, then in PBS alone, and subsequently mounted in a mixture of PBS and glycerol (1:1) for confocal laser scanning microscopy analysis.

### Fluorescence recovery after photobleaching

FRAP experiments were performed on living transverse vibratome stem sections of ash (*n*=13) and poplar (*n*=13) incubated with FM 4-64FX at room temperature for 15 min and washed in distilled water. The protocol for FM 4-64FX intercellular transport in wood is based mostly on GAP-FRAP analyses (Abbaci *et al*., 2007; Stasiak *et al*., 2020).

To monitor changes in fluorescence, three regions of interest (ROI) were manually outlined on a pre-scanned image: (i) around the border of a whole target cell (outlined in white), (ii) around an unbleached reference cell (outlined in green), and (iii) in a region outside the cells to measure background fluorescence intensity (outlined in blue). The baseline scans were excited using a 561 nm laser. The bleaching procedure was performed for 30 s using a 405 nm laser with the power adjusted to 100%. Subsequently, the fluorescence recovery was recorded during two-dimensional time-lapse repeated scanning using the same settings as those used for the baseline scans.

At each time point, the fluorescence intensity values of ROI 1were divided by those of ROI 2 and plotted as a function of time to obtain the FRAP recovery curves. These curves were fitted to a single exponential function:


$$\begin{array}{l} F\left(t\right)=Fb+Fr\left(1-e^{-t/\text{rmtau}}\right) \end{array}$$

where *t* is the time after photobleaching, *Fb* is fluorescence intensity estimated immediately after photobleaching, *Fr* is the asymptotic value to which the fluorescence intensity tends, and τ is the time at which fluorescence intensity recovers 50% of its initial fluorescence measured before photobleaching. To better fit the FRAP curves to the function, a transfer constant *k*=1/τ was calculated (Abbaci *et al*., 2007). Plotting intensity values and fitting the function were performed using GNU Octave version 6.2.0 (The Octave Project Developers). Knowing the initial fluorescence before photobleaching *F*_*0*_, the mobile fraction (*R*) was calculated:


$$\begin{array}{l} R=\frac{Fr-Fb}{F_{0}-Fb}\times 100 \end{array}$$

### RNA isolation and quantitative real-time PCR

Wood fragments (approximately 350 mg) were isolated from poplar plants (adjacent tissues were carefully removed) and ground using a TissueLyser II (Qiagen). Total RNA was extracted using a Ribospin^TM^ Plant kit (GeneAll, South Korea) and treated with a DNA-free^TM^ Kit (Ambion) to remove any residual DNA. Reverse transcription was performed using 0.5 μg total RNA and a High-Capacity cDNA Extraction Kit (A&A Biotechnology, Poland). Quantitative real-time PCR (qRT–PCR) analyses were performed using a LightCycler480 (Roche, Germany) and Real-Time 2× PCR Master Mix SYBR version B (A&A Biotechnology, Poland), with a final primer concentration of 0.5 µM. All experiments were conducted in triplicate. The primer sequences and amplification conditions were as follows: denaturation for 1 min (95 °C), 45 cycles of denaturation for 10 s (95 °C), amplification for 10 s (50–60 °C), elongation for 20 s (72 °C), and cooling for 30 s (40 °C) (Supplementary Table S1). Melting curve analysis was performed to test the specificity of primers, and the *UBIQUITIN* (*UBCE2a*, potri.019g039200) and *ELONGATION FACTOR 1-b* (potri.001G224700, *EF1b*) genes were used as reference; ∆∆Ct values were calculated to quantify the data. The expression of the selected genes [*SUCROSE TRANSPORTER 3* (potri.019g085800, *SUT3*), *SUCROSE TRANSPORTER 4* (potri.002g106900, *SUT4*), *CLATHRIN HEAVY CHAIN* (potri.008g070600, *CHC*), *CLATHRIN LIGHT CHAIN* (potri.004g040100, *CLC*), *REMORIN 1.4* (potri.001g107000, *REM1.4*), and *REMORIN 6.1* (potri.008g144300, *REM6.1*)] analysed in this study has been previously confirmed in poplar wood (Sundell *et al*., 2017).

### Microscopy

Vibratome sections were analysed using an epifluorescence microscope BX60 (Olympus Co., Japan) with a digital camera (DP73, Olympus) or by confocal laser scanning microscopy (CLSM), using a FluoView 100 (Olympus) and NikonA1Rsi with Nikon NIS Elements AR software. The excitation (ex) and emission (em) wavelengths were as follows: ex 488 nm and em 525/50 nm for FM 1-43FX; ex 561 nm and em 595/50 nm for FM 4-64FX and 3TR. TEM was performed using Zeiss 900 EM (80 kV) and Hitachi H-800 (150 kV) systems.

### Statistical analysis

The location of thresholds, abrupt changes, or discontinuities in biological processes under investigation can be estimated using change-point models (Lavielle and Lebarbier, 2001; Beckage *et al.*, 2007). Therefore, multiple change-point analyses based on Bayesian interference and Markov Chain Monte Carlo simulation were performed in R (R Core Team, 2015) to compare intra-annual changes in NSC content and FM 4-64FX dye abundance. First, the linear regression model must be fitted to the data in intervals (segmented regression, where each segment can be represented by a different model). To find a suitable model using the ‘mcp’ package, the number of segments it should consist of (which can be done e.g. on the basis of a priori knowledge or by analysing a plot) and the form of the model (e.g. whether the regression model consist of intercepts only, or whether models need to stick together) were specified. Subsequently, the points where the next segment begins (the so-called change-points) and the coefficients of each model segment are determined by the algorithm. Coefficient models were assessed based on Bayesian inferences. A univariate distribution was considered as the initial location of the change-points (a priori distribution), and their posterior distribution was estimated using the Markov Chain Monte Carlo (MCMC) simulation method. The R code used to analyse the change-points is provided in Supplementary Dataset S1. Other statistical analyses (with α=0.05) were performed using Statistica version 13 (TIBCO Software Inc., 2017). ANOVA with post-hoc Tukey’s test was used to compare differences in dye abundance between the four seasons in ash and maple. The Kruskal–Wallis test was used to analyse seasonal changes in dye abundance in poplar, differences in gene expression levels, and changes in soluble sugars and sugar alcohols. The Mann–Whitney U-test was used to determine the impact of TA23 on VACs and nonVACs in all tree species. A two-sample *t*-test was used to analyse the results of the FRAP experiments.

## Results

### Living cells of ash, maple, and poplar wood

**Fig. 1.**
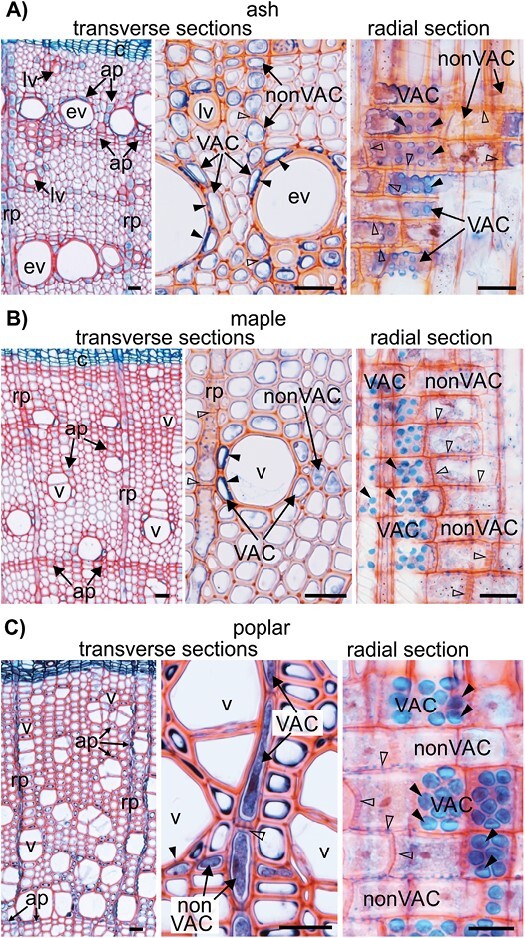
Wood anatomy of *Fraxinus excelsior* (ash), *Acer pseudoplatanus* (maple), and *Populus tremula* × *tremuloides* (poplar). (A) Structure of ring-porous wood on transverse and radial sections of ash stem. Earlywood vessels can be seen that are clearly larger than latewood vessels (left panel), adjoined by XPCs, including VACs with distinct contact pits (black arrowheads) and nonVACs with simple pits (empty arrowheads) (middle and right panels). (B, C) Structure of diffuse-porous wood on transverse and radial sections of maple (B) and poplar (C) stems. Vessel elements of similar dimensions (left panels) are surrounded by XPCs, including VACs with distinct contact pits (black arrowheads) and nonVACs with simple pits (empty arrowheads) (middle and right panels). The functional types of VACs and nonVACs formed by axial parenchyma cells are shown in the middle panels for ash (A) and maple (B) wood, and those formed by ray parenchyma are shown in the right panels. Most of the XPCs in poplar wood (C) belong to the ray system; individual axial parenchyma cells that function as VACs and nonVACs are shown in the middle panel. All sections were viewed under bright-field microscopy. Bars=20 µm. ap, axial parenchyma; c, cambium; ev, earlywood vessel; lv, latewood vessel; nonVAC, non-vessel-associated cell; rp, ray parenchyma; v, vessel; VAC, vessel-associated cell; XPC, xylem parenchyma cell.

In the wood of ash (ring-porous type) and maple and poplar (diffuse-porous type), both VACs and nonVACs were determined among the ray and axial xylem parenchyma (Fig. 1; Supplementary Fig. S2). VACs were characterized by the presence of contact pits facilitating direct contact with vessel elements. In addition, simple pits were detected between VACs and nonVACs, and between nonVACs (Fig. 1). Ash and maple have abundant ray and axial parenchyma (Fig. 1; Supplementary Fig. S2), whereas poplar has xylem parenchyma composed mainly of uniseriate rays and the axial parenchyma is scarce.

### Intra-annual changes in cambial activity and wood formation of ash and maple

**Fig. 2.**
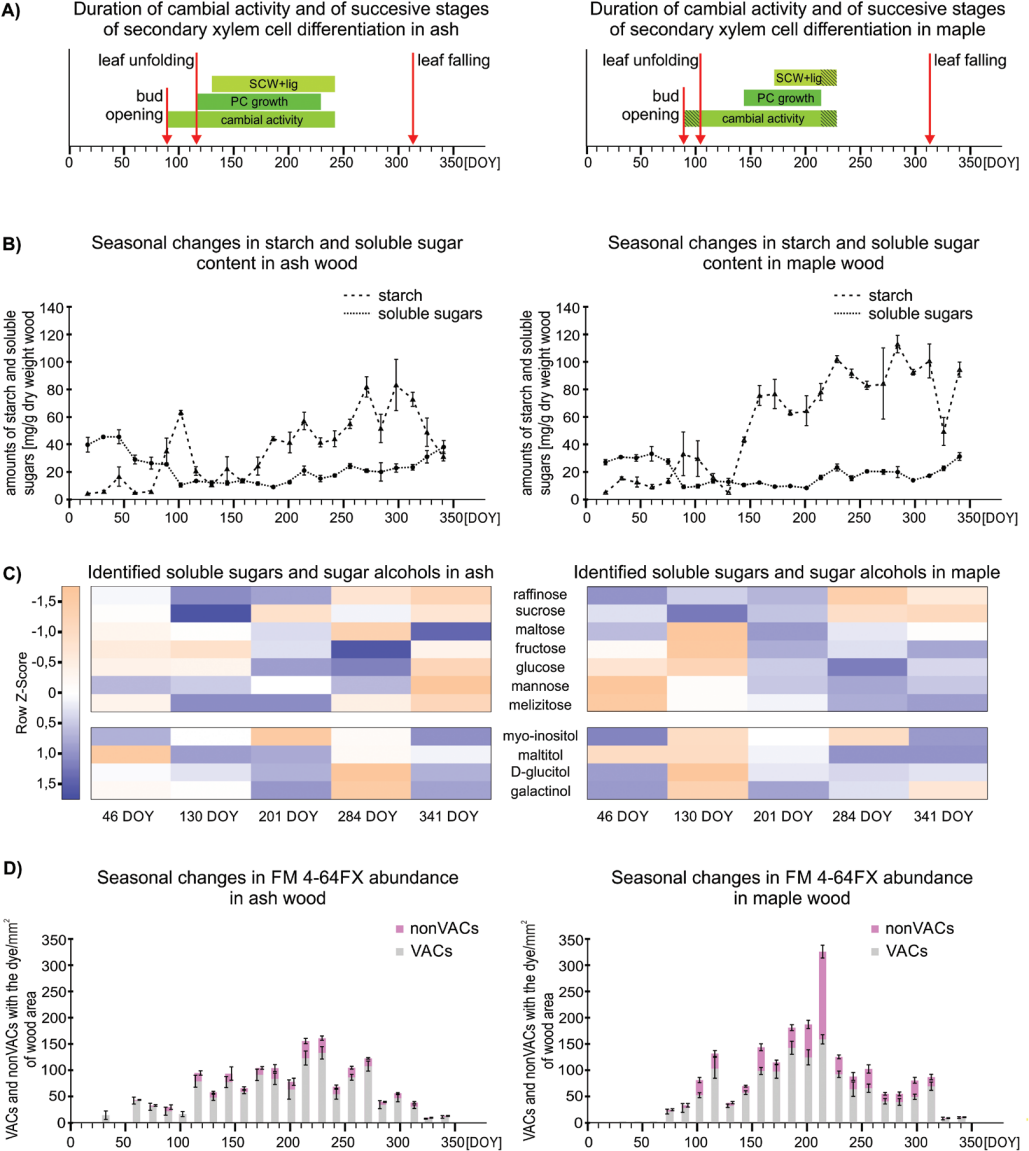
Seasonal changes in cambial activity, wood formation processes, and non-structural carbohydrate content of ash and maple trees. (A) Cambial activity and stages of wood element differentiation in ash and maple trees during the year. DOY, day of year; PC, post-cambial growth; SCW+lig, secondary cell wall formation and lignification. Hatched regions indicate differences in the onset and completion of the consecutive stages between the analysed branches. Vertical arrows indicate the timing of the selected phenological events. (B) Annual variations in starch and soluble sugar content in ash and maple wood detected using a colorimetric method; data presented are means ±SE. (C) Metabolomic data (GC-MS experiment). Heatmap analysis of sugars and sugar alcohols in maple (*n*=6) and ash (*n*=6) wood for each analysed time point. The presented metabolites are differentially abundant (*P*<0.05) according to the Kruskal–Wallis test (for quantitative data and their statistical relevance, see Supplementary Fig. S5). The colour gradient illustrates the *Z*-scores of seven sugars and four sugar alcohols identified in maple and ash wood at five different time points (46, 130, 201, 284, and 341 DOY). The presented data were calculated as mean-centred normalized intensity values divided by the SD for each metabolite. Variations in the relative abundance of metabolites are displayed on a colour gradient from blue (minimal abundance) to pink (maximal abundance). (D) Seasonal changes in FM 4-64FX abundance in vessel-associated cells (VACs) and non-vessel-associated cells (nonVACs) during 2016 in ash and maple wood. Data presented are means ±SE.

During winter, in ash and maple, the cambial zone comprised only two to four radially flattened cells (Supplementary Fig. S3A). The onset of bud swelling and opening started in spring at 89 DOY and proceeded for 4 weeks in ash and 2 weeks in maple, until the completion of leaf expansion (Fig. 2A). In both species, the first divisions in the cambium (Supplementary Fig. S3A) were observed in parallel to bud opening (89 DOY), but more frequent cambial divisions were visible at 104 DOY. Cambial activity was not detected at 242 DOY in ash and at 229 DOY in maple (Fig. 2A). The onset of post-cambial growth in ash began in spring at 116 DOY, soon after cambial activation, and preceded the differentiation of the first earlywood vessels from overwintering cambial derivatives (130 DOY; Fig. 2A; Supplementary Fig. S3A). Post-cambial growth in maples began in summer at 144 DOY (Fig. 2A), and the deposition of the secondary cell wall, which coincided with vessel formation, began at 158 DOY (Fig. 2A; Supplementary Fig. S3A). Differentiation processes in wood and secondary cell wall deposition (Supplementary Fig. S3A) were observed for the last time at 242 DOY in ash and 229 DOY in maple (Fig. 2A). Leaf fall in both species started at 313 DOY and leaves were completely lost at 326 DOY after the first frost (Fig. 2A; Supplementary Fig. S1A).

### Seasonal changes in NSC content in maple and ash wood

Starch is a substantial form of accumulated NSC deposited in both the ray and axial parenchyma (Supplementary Fig. S3C), and its content reflects seasonal variations in both ash and maple wood (Fig. 2B). In spring, starch amounts temporarily increased (at 102 DOY in ash and 89–102 DOY in maple) but then declined to their minimal values at 130 DOY. Subsequently, the starch content increased substantially in summer, reached its highest value in autumn, and then decreased again during winter (Fig. 2B; Supplementary Fig. S4A). The starch content in summer and autumn was higher in maple than in the ash. In contrast, the transient peak of starch content in spring appeared later, but was higher, in ash than in maple (Fig. 2B).

The soluble sugar content in ash and maple wood also showed seasonal variation. The highest concentrations were detected during winter (Fig. 2B). Before bud opening, sugar content markedly declined, and then continuously increased from summer to winter in both species (Fig. 2B; Supplementary Fig. S4A). Substantial amounts of soluble sugars (sucrose, fructose, maltose, glucose, mannose, and raffinose) and sugar alcohols (myo-inositol, galactinol, and d-glucitol) were identified in the wood of both species. Melizitose and maltitol were also detected in ash wood. The amounts of specific sugars and sugar alcohols exhibited clear seasonal variations (Fig. 2C; Supplementary Fig. S5). Some accumulated in greater amounts during autumn and winter (e.g. mannose and raffinose in ash and maple, sucrose in maple), and others during spring (e.g. fructose in ash and maple) or summer (e.g. myo-inositol in ash) (Fig. 2C; Supplementary Fig. S5).

### Seasonal changes in FM 4-64FX dye abundance in angiosperm wood

By loading FM 4-64FX dye into ash and maple trees used for the anatomical and NSC studies described above, we quantified the number of XPCs (namely, VACs and nonVACs) with the dye signal in relation to wood area for each time point and species. In both species, the FM 4-64FX dye signal was mainly localized in the VACs, but it was also detected in the nonVACs, and the dye abundance varied throughout the year (Fig. 2D; Supplementary Fig. S6). During winter dormancy, the FM 4-64FX abundance was minimal and ranged from 9.94 ± 1.42 to 43.62 ± 6.93 XPCs in ash and 8.24 ± 3.24 to 86.28 ± 12.05 XPCs in maple per 1 mm^2^ of wood area (Fig. 2D). At 116 DOY, when leaves were fully unfolded, dye abundance greatly increased during spring (Fig. 2D; Supplementary Fig. S4B), and reached 94.04 ± 15.37 in ash and 131.06 ± 26.15 XPCs in maple per 1 mm^2^ of wood area. Furthermore, it was clearly reduced in maple at 120 DOY (Fig. 2D) after the last spring frost (Supplementary Fig. S1A). During summer, dye abundance varied from 66.04 ± 16.36 to 161.54 ± 14.42 XPCs in ash and from 69.49 ± 4.34 to 326.36 ± 14.87 XPCs in maple per 1 mm^2^ of wood area (Fig. 2D). The highest values were observed between DOY 214 and DOY 229 for ash, and between DOY 186 and DOY 214 for maple (Fig. 2D). In autumn, when all wood cells were mature, dye abundance was substantially lower compared with summer data (Fig. 2D; Supplementary Fig. S4B), and at 284 DOY dropped to 40.18 ± 8.49 XPCs in ash and to 54.48 ± 10.47 XPCs in maple per 1 mm^2^ of wood area (Fig. 2D; Supplementary Fig. S6). After the first winter frost, at 326 DOY (Supplementary Fig. S1A), dye abundance was substantially reduced in both species (Fig. 2D).

### Involvement of varied transport mechanisms is season and species dependent

**Fig. 3.**
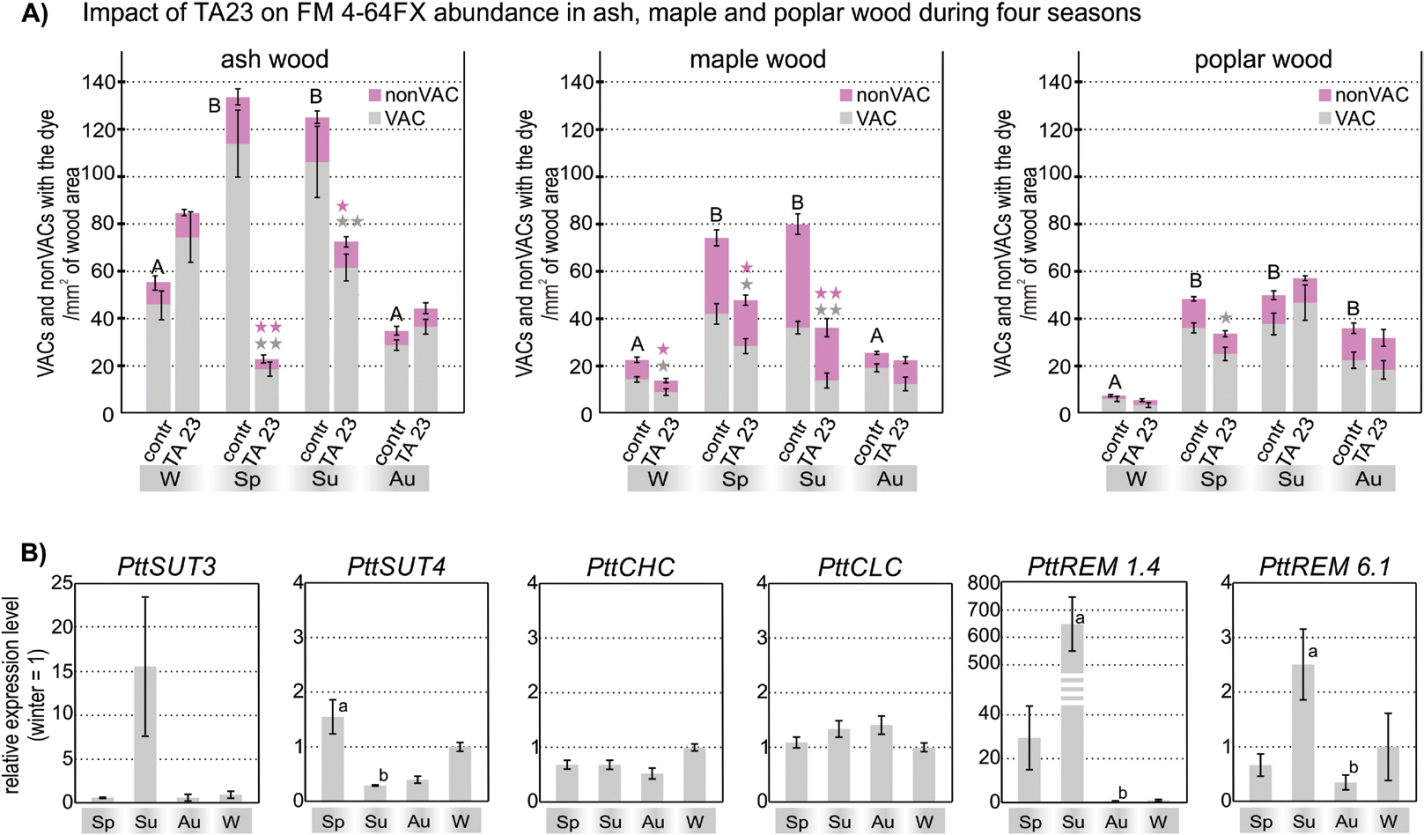
Season- and species-dependent involvement of varied transport mechanisms in angiosperm wood. (A) Annual differences in FM 4-64FX abundance in xylem parenchyma cells (XPCs) [vessel-associated cells (VACs) and non-vessel-associated cells (nonVACs)] of ash, maple, and poplar wood of control trees (contr) and those subjected to the endocytic inhibitor tyrphostin A23 (TA23). Results collected from at least 16 (for winter), 24 (for spring), 34 (for summer), and 18 (for autumn) areas of wood obtained from at least two independent loading experiments for each season and species (W, winter; Sp, spring; Su, summer; Au, autumn) are shown. Data presented are means ±SE. Different letters indicate significant differences in FM 4-64FX abundance in XPCs between the seasons. Significant differences upon TA23 treatment relative to the control (within the particular season) are shown with asterisks (*α=0.05, **α=0.01; two-sample *t*-test): differences in the dye transport into VACs are indicated with grey asterisks, and differences in the dye transport into and between nonVACs are indicated with purple asterisks. (B) Changes in the expression levels of genes encoding sucrose transporters (*PttSUT3*, *PttSUT4*), clathrin-related genes (*PttCHC*, *PttCLC*), and remorins (*PttREM1.4*, *PttREM6.1*) based on the expression of the reference gene *UBCE2a*. Results are from poplar branches (*n*=34) that were separately collected during the four seasons. Significant differences are indicated with different letters [*PttSUT4 P*=0.026820, *PttREM1*.*4 P*=0.022029, *PttREM6*.*1 P*=0.008422; (Kruskal-Wallis test, α=0.05)]. Data presented are means ±SE.

CME plays an important role in FM 4-64FX transport from the vessel elements into the VACs in the ring-porous wood of ash and the diffuse-porous wood of maple and poplar (Słupianek *et al*., 2019). Therefore, we analysed seasonal and species variations under the effect of TA23, a CME inhibitor, on FM 4-64FX abundance in the VACs and nonVACs of all three species during winter, spring, summer, and autumn (Fig. 3A). In the control (untreated) ash, maple, and poplar trees, FM 4-64FX was localized to both VACs and nonVACs, and the dye abundance was the highest during the vegetative season (spring and summer) (Fig. 3A; Supplementary Figs S6, S7). This high FM 4-64FX abundance in the vegetative season was significantly different compared with winter and autumn samples in ash and maple trees, and significantly different compared only with winter in poplar (Fig. 3A). Upon TA23 treatment, dye abundance in both VACs and nonVACs decreased significantly during spring and summer in ash, and during winter, spring, and summer in maple. Notably, dye abundance in poplar decreased significantly only in VACs during spring, and no significant difference was detected in nonVACs compared with the control (Fig. 3A).

A significant decrease in FM4-64FX abundance in VACs upon TA23 treatment in only some seasons suggests the involvement of mechanisms other than CME. Therefore, during the four seasons, we examined the transcript levels of several genes, including *SUT3* and *SUT4* as examples of genes coding for membrane transporters (Payyavula *et al*., 2011; Mahboubi *et al*., 2013); *CHC* and *CLC*, which code for proteins that are essential for CME-associated processes (Baisa *et al*., 2013); and *REM1.4* and *REM6.1*, which code for plasma membrane proteins localized in membrane microdomains, to study clathrin-independent endocytosis (Fan *et al*., 2015; Gronnier *et al*., 2017) (Fig. 3B; Supplementary Table S1). *PttSUT3* showed elevated transcript levels in summer compared with spring, and *PttSUT4* was significantly increased in spring compared with summer (Fig. 3B). Transcript levels of *PttCHC* and *PttCLC* were generally similar across all the seasons (when *EF1b* rather than *UBCE2a* was used as the reference gene, *PttCLC* transcript levels were significantly higher in autumn compared with winter), and *PttREM1*.*4* and *PttREM6*.*1* significantly increased during summer compared with autumn/winter (Fig. 3B; Supplementary Fig. S8). Notably, the increase in the transcript levels of *PttREM1.4* seemed to be particularly strong during summer (Fig. 3B).

### Transport of membrane dyes between XPCs in ash and poplar

The localization of FM 4-64FX in both VACs and nonVACs and the reduction of dye abundance in nonVACs only when transport to VACs was hampered (Figs 2, 3; Supplementary Figs S6, S7) suggest that molecules internalized into VACs via endocytosis are transported farther between XPCs by a different mechanism. Thus, we further investigated transport processes using ash (ring-porous) and poplar (diffuse-porous) wood.

**Fig. 4.**
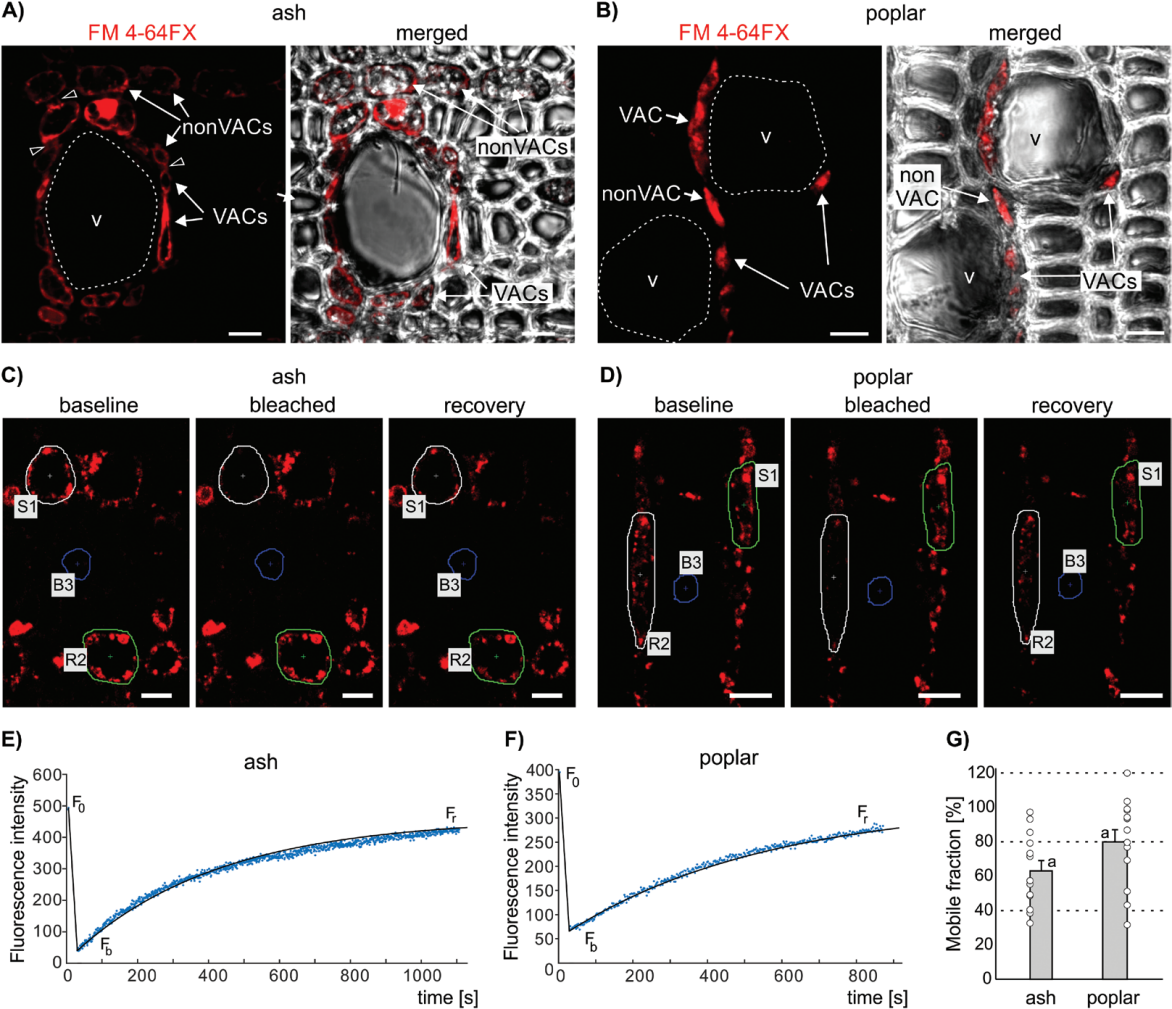
Cell-to-cell transport of FM 4-64FX dye in ash and poplar wood. (A, B) Localization of FM 4-64FX in vessel-associated cells (VACs) and non-vessel-associated cells (nonVACs) in ash (A) and poplar (B) wood. The panels on the right show the same images merged with transmitted light images. Empty arrowheads indicate simple pits. The lumens of the selected vessel elements (v) are outlined. Bars=10 µm. (C–G) Fluorescence recovery after photobleaching (FRAP) experiments. (C, D) Localization of FM 4-64FX dye before photobleaching (baseline), immediately after bleaching, and at the end of FRAP showing recovery in ash (C) and poplar (D). The target cell subjected to photobleaching is outlined with white (S1), an unbleached reference cell is outlined with green (R2), and the blue outline marks the region where the fluorescence intensity of the background was measured (B3). Bars=10 µm. (E, F) Representative FRAP curves fitted to an exponential function (black line) for ash (E) and poplar (F), with the fluorescence intensity in the target cell at baseline (F_0_), after bleaching (F_b_), and after recovery (F_r_) indicated. (G) Mean ±SE values of the mobile fraction of FM 4-64FX transported between xylem parenchyma cells of ash and poplar.

Using CLSM, we analysed the subcellular localization of the membrane dyes FM 1-43FX and FM 4-64FX after loading them into the vascular system. In ash and poplar wood, fluorescent signals were localized in the plasma membranes and various endocytic compartments in both VACs and nonVACs (Fig. 4A, B; Supplementary Fig. S9A). Importantly, the dyes were also visible in the simple pits connecting neighbouring living parenchyma cells (Fig. 4A; Supplementary Fig. S9A). Next, we estimated *in vivo* mobility of FM 4-64FX between XPCs by using FRAP (Fig. 4C–G). The changes in fluorescence intensity in the target cells after photobleaching (Fig. 4C, D; Supplementary Videos S1, S2) were visualized as recovery curves fitted to an exponential function (Fig. 4E, F) and were used to calculate the mobile fraction (R) of FM 4-64FX during transport between the XPCs. The mean R value was determined as 63.1% ±5.96% for ash and 80.4% ±7.13% for poplar, confirming *in vivo* cell-to-cell mobility of FM 4-64FX between living wood cells of the analysed species. No significant differences in the calculated mean R values were found between the species (Fig. 4G).

### Intercellular transport of 3TR ‘trapped’ in the endomembrane system

**Fig. 5.**
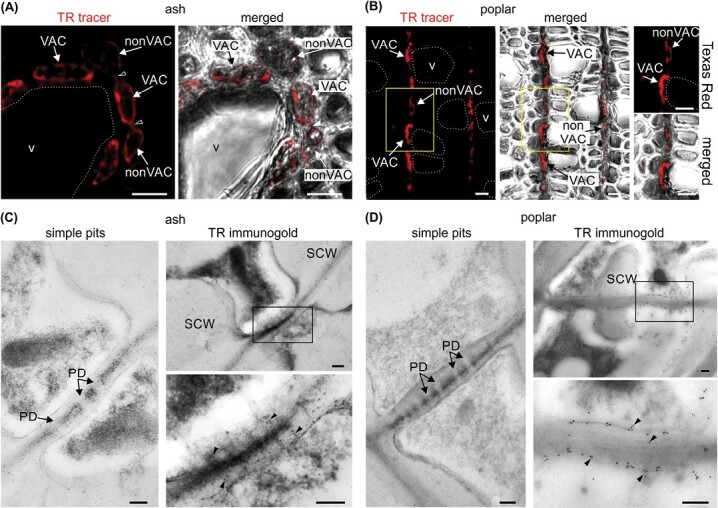
Cell-to cell transport of Texas Red 3 kDa (3TR) dye between xylem parenchyma cells (XPCs) of ash and poplar wood. (A, B) Confocal images showing the localization of 3TR dye in vessel-associated cells (VACs) and non-vessel-associated cells (nonVACs), and in simple pits (empty arrowheads) connecting neighbouring XPCs in ash (A) and poplar (B) wood. The lumens of the selected vessel elements (v) are outlined. The panels on the right show the same images merged with transmitted light images. Regions marked with yellow frames are magnified (right). Bars=10 µm. (C, D) TEM images of ash (C) and poplar (D) XPCs showing numerous simple plasmodesmata (PD) gathered in simple pits (left) and TR immunogold (right). The simple pit regions framed in the upper images are magnified in the lower images. Black arrowheads indicate 10 nm gold particles attached to the primary anti-TR antibody. SCW, secondary cell wall. Bars=200 nm.

To study the involvement of plasmodesmata in dye transport within the endomembrane system at both the cellular and ultrastructural levels, 3TR, an endocytic large molecular mass dye, was used. 3TR was first visualized in wood by CLSM. When loaded into ash and poplar, 3TR was localized in both the VACs and neighbouring nonVACs (Fig. 5A, B), and was visible in the simple pits connecting XPCs (Fig. 5A). These results suggest that, after internalization into VACs, 3TR can move intercellularly into nonVACs via simple pits in a manner similar to FM 4-64FX, for which cell-to-cell transport was demonstrated by FRAP (Fig. 4C–G). In both species, the simple pits connecting the XPCs contained numerous plasmodesmata, which were indirectly visualized by callose immunolocalization (Supplementary Fig. S9B) and directly confirmed by ultrastructural studies using TEM (Fig. 5C, D). Finally, on transverse ultrathin sections obtained from ash and poplar loaded with 3TR dye, we performed immunogold studies with an anti-TR primary antibody and secondary antibody conjugated to 10 nm gold particles. Gold particles corresponding to 3TR were localized in the neck and central parts of the plasmodesmata (Fig. 5C, D).

## Discussion

The xylem parenchyma is a key component of angiosperm wood (Couturier *et al*., 2010; Secchi *et al*., 2011; Hartmann and Trumbore, 2016; Morris *et al*., 2020). Although crucial progress has been made in our understanding of transport mechanisms in trees, in-depth analyses linking the annual dynamics of angiosperm trees to these transport mechanisms are lacking.

### Seasonal changes in dye abundance relate to the amount of living cells in wood

The significant increase in dye abundance in wood of both ring- and diffuse-porous species coincided with the resumption of growth in spring, during which cambial division activity restarted, NSCs that had been stored for winter were depleted, and the first leaves appeared. Elevated dye abundance was maintained during summer, when cambial divisional activity and differentiation of xylem derivatives occurred (Fig. 2). These observations are consistent with the commonly accepted understanding that the greatest transport intensity occurs during the vegetative growth season (Tixier *et al.*, 2017; Kuroda *et al.*, 2020; Ray and Savage, 2021). In autumn and winter, starch and soluble sugars (e.g. mannose and raffinose) accumulate for energy storage and also act as cryoprotectants and stress-induced signals (Kasuga *et al*., 2007; Egert *et al*., 2013). A significant reduction in dye abundance was observed in both ring- and diffuse-porous species during this time (Fig. 2D). This may be the outcome of decreased axial conduction due to cavitation of vessel elements (Utsumi *et al*., 1998, 1999; Jaquish and Ewers, 2001) or reduced endocytosis activity in the VACs after exposure to low temperatures (Deng *et al.*, 2015; Wang *et al*., 2020).

Given the role of the parenchyma in cell-to-cell transport within wood and in linking the cambium with the adjoining internal (xylem) and external (phloem, bark) tissues (Chaffey and Barlow, 2001; Pfautsch *et al*., 2015; Secchi *et al*., 2017), it is plausible that greater amounts of XPCs are responsible for the higher dye abundance found in ash and maple than in poplar wood (Figs 1, 2D, 3A). This explanation is in line with the findings of Treydte *et al*. (2021) that a larger amount of ray parenchyma increases the capacity for radial and circumferential water transport in *Eucalyptus sideroxylon* trunks, providing evidence of the regulatory role of living parenchyma in xylem hydraulic efficiency and safety (De Baerdemaeker *et al*., 2018; Kiorapostolou *et al.*, 2019; Aritsara *et al*., 2021).

### Plasmodesmata facilitate transport of molecules ‘trapped’ in the endomembrane system between living XPCs

**Fig. 6.**
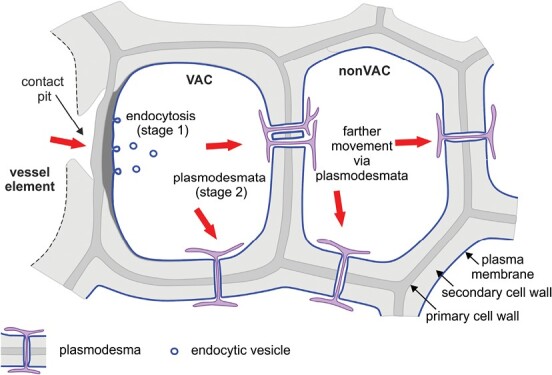
Proposed model showing a two-stage process of cell-to-cell transport in angiosperm wood. Molecules are first unloaded from vessel elements into living vessel-associated cells (VACs) via endocytosis (stage 1) and then move farther from the VACs into neighbouring non-vessel-associated cells (nonVACs), via plasmodesmata (stage 2). Red arrows indicate the directions of transport in each stage. The lumen of a vessel element is outlined with dashed black lines.

In this study, we identified two important stages of cell-to-cell transport of dye in angiosperm wood (Fig. 6). First, molecules are internalized into the VACs via endocytosis (stage 1), followed by their continued transport through plasmodesmata into and between the nonVACs (stage 2). The FRAP studies confirmed the ability of FM 4-64FX dye to move between XPCs at similar rates in ash and poplar, suggesting the universality of this phenomenon among ring- and diffuse-porous trees (Fig. 4C–G). Furthermore, using CLSM and TEM, we demonstrated that the endocytic marker 3TR, which is characterized by a high molecular mass, was transported from VACs to nonVACs and could be detected in plasmodesmata joining living wood cells (Fig. 5). These results indicate, for the first time, that markers ‘trapped’ in the endomembrane system, even those of a high molecular mass, can be transported via plasmodesmata in angiosperm wood. Therefore, we hypothesized that large endogenous molecules present in wood that are localized in the plasma membrane and elements of the endomembrane system can be transported intercellularly through plasmodesmata, a phenomenon previously observed only for plant viruses and bacteria (Carluccio *et al.*, 2014; Feng *et al.*, 2016; Ishikawa *et al.*, 2017; Li *et al.*, 2021a), and for the SHORT-ROOT protein in Arabidopsis roots (Spiegelman *et al*., 2019). Transport of large molecules via plasmodesmata may occur in the membrane or lumen of desmotubules (Guenoune-Gelbart *et al*., 2008; Barton *et al*., 2011) and can be regulated by specific plasmodesmata-associated proteins (Kriechbaumer *et al.*, 2015; Brault *et al.*, 2019; Chen *et al.*, 2021). However, the exact mechanism of transport of molecules via plasmodesmata between living XPCs of angiosperm wood requires further investigation.

### Transport in wood is regulated by mechanisms that appear to be species and/or season dependent

Our data suggest that not only are the two stages of cell-to-cell transport in wood regulated by various mechanisms, predominantly CME in stage 1 (Słupianek *et al*., 2019) and plasmodesmata in stage 2, but also the involvement of these mechanisms is species and season dependent. We found that TA23, a CME inhibitor, significantly lowered the dye abundance in the VACs during spring and summer in ash, in winter, spring, and summer in maple, and in spring in poplar. In contrast, TA23 mostly did not affect the second stage of dye transport. The presence of dye was decreased significantly in the nonVACs of only ash and maple, where FM 4-64FX transport to VACs was also hampered (Fig. 3A). Thus, it is plausible that CME does not play a role during stage 2, and that reduced dye transport during stage 1 upon TA23 treatment inextricably led to reduced dye transport during stage 2. In this study, the expression of representatives of two different groups of remorin genes (*PttREM1.4* and *PttREM6.1*), which are functional elements of the plasma membrane microdomain regions involved in clathrin-independent processes (Gronnier *et al*., 2017), reinforces our hypothesis that microdomain-associated endocytosis, in addition to CME, is involved in the uptake of molecules (mostly those of higher molecular mass) into VACs (Słupianek *et al*., 2019). Importantly, the transcript levels of *PttREM1.4* and *PttREM6.1* were elevated in spring and summer (Fig. 3B; Supplementary Fig. S8), suggesting annual variations in the involvement of clathrin-independent endocytic transport mechanisms in wood. During the vegetative season, the abundance of FM 4-64FX dye in poplar wood reached its highest value (Fig. 3A; Supplementary Fig. S7); thus, the cooperation of different endocytic pathways, both clathrin mediated and clathrin independent, may be crucial for enhancing molecular uptake from the xylem sap into the VACs. The endocytic transport pathways in cells are complemented by exocytosis (Reynolds *et al*., 2018; Zhang *et al*., 2019). Thus, it is easy to assume that exocytosis also occurs in the VACs and is involved in the release of molecules into the xylem sap (van Bel, 2021). This assumption is supported by the identification of exosomes in the pit formation sites of spruce and birch wood (Chukhchin *et al*., 2020). However, further studies are required.

In addition to the endo- and exocytic pathways, cell-to-cell transport in wood is based on membrane transporters that facilitate the translocation of sugars and amino acids between the apoplast and living xylem parenchyma cells (Dobbelstein *et al*., 2019; Gratz *et al.*, 2021; Zhang *et al.*, 2021). Sucrose transporters mediate sucrose import from the apoplasmic space, and act seasonally in *Quercus robur*, *Fagus sylvatica*, and *Juglans regia* (Decourteix *et al*., 2006; Dobbelstein *et al*., 2019). In poplars, the involvement of sucrose transporters (*PttSUT3* and *PttSUT4*) in carbon allocation has been demonstrated, but only in samples harvested during the vegetative season. Thus, we analysed the expression levels of *PttSUT3* and *PttSUT4* throughout the year and observed elevated expression levels in the vegetative season (*PttSUT3* in summer, and *PttSUT4* in spring) (Fig. 3B; Supplementary Fig. S8), when plant activity and the processes of xylem derivative formation and maturation were the most intense. This is in agreement with the presumed role of *PttSUT4* in sucrose utilization and transport through the stem (Payyavula *et al*., 2011), and the involvement of *PttSUT3* in sucrose import from the apoplasm to developing fibres (Mahboubi *et al.*, 2013). Moreover, our results support the assumption that intensive cell-to-cell transport in wood is partially mediated by membrane transporters in addition to endocytosis and plasmodesmata.

### Conclusions

Our findings elucidate the mechanisms and seasonal dynamics of cell-to-cell transport in wood, and highlight the importance of living XPCs in angiosperm trees. The four crucial findings from our study are: (i) plasmodesmata are involved in intercellular transport of the dyes internalized from vessel elements to VACs; thus, the dye transport is a two-stage process (stage 1: from vessel elements to VACs via endocytosis; stage 2: from VACs to nonVACs via plasmodesmata); (ii) in angiosperm wood, even large-molecular-mass dyes ‘trapped’ in the endomembrane system can move between living cells; (iii) intra-annual changes in the abundance of endocytic membrane dye are present in wood and are characteristic of both ring- and diffuse-porous wood types; and (iv) the contribution of different transport mechanisms in angiosperm wood, including endocytosis, membrane transporters, and plasmodesmata, is dependent on the wood type and season.

## Supplementary data

The following supplementary data are available at *JXB* online.

Fig. S1. Temperature and precipitation data in Wroclaw during 2016 and between 2017 and 2020.

Fig. S2. Anatomy of ash, maple, and poplar wood, corresponding to Fig. 1.

Fig. S3. Anatomical studies of ash and maple wood, corresponding to Fig. 2.

Fig. S4. Multiple change-point analysis in mean of seasonal changes in starch and soluble sugars, and in FM 4-64FX abundance of ash and maple trees, corresponding to Fig. 2B, D.

Fig. S5. Annual variations in the content of soluble sugars and their compounds in ash and maple wood, corresponding to Fig. 2C.

Fig. S6. Seasonal differences in FM 4-64FX localization in ash and maple wood during the four seasons, corresponding to Figs 2D, 3A.

Fig. S7. Seasonal differences in FM 4-64FX localization in poplar wood during the four seasons, corresponding to Fig. 3A.

Fig. S8. Changes in the expression levels of sucrose transporters, clathrin-related genes, and remorins; results based on *EF1b* reference gene corresponding to Fig. 3B.

Fig. S9. Localization of FM dyes and callose in the xylem parenchyma cells of ash and poplar wood, corresponding to Figs 4, 5.

Table S1. Sequences of oligonucleotides used for qRT–PCR, corresponding to Fig. 3B.

Dataset S1. The R code used to analyse the change points in intra-annual changes in NSC content or FM 4-64FX dye abundance in ash and maple wood.

Video S1. FRAP in *Fraxinus excelsior* (ash).

Video S2. FRAP in *Populus tremula* × *tremuloides* (poplar).

erad469_suppl_Supplementary_Figures_S1-S9_Tables_S1_Datasets_S1

erad469_suppl_Supplementary_Videos_S1

erad469_suppl_Supplementary_Videos_S2

## Data Availability

All data supporting the findings of this study are available within the paper and within its supplementary data published online.

## Abbreviations

3TR: Texas Red 3 kDa
CLSM: confocal laser scanning microscopy
CME: clathrin-mediated endocytosis
DOY: day of the year
FRAP: fluorescence recovery after photobleaching
nonVAC: non-vessel-associated cell
XPC: xylem parenchyma cell
NSC: non-structural carbohydrate
PBS: phosphate-buffered saline
TA23: tyrphostin A23
VAC: vessel-associated cell
