# Supplementary material for: Seasonal dynamics of cell-to-cell transport in angiosperm wood
Source: J Exp Bot. 2023 Nov 23;75(5):1331–46. doi: 10.1093/jxb/erad469 (PMC10901208; doi:10.1093/jxb/erad469)
Supplement: erad469_suppl_Supplementary_Figures_S1-S9_Tables_S1_Datasets_S1 [file erad469_suppl_supplementary_figures_s1-s9_tables_s1_datasets_s1.pdf]

Supplementary Data

Article title: *Seasonal dynamics of cell-to-cell transport in angiosperm wood*

Authors: *Aleksandra Słupianek[1], Elżbieta Myśkow[1], Anna Kasprowicz-Maluśki[2], Alicja Dolzblasz[1], Roma Żytkowiak[3], Magdalena Turzańska[1], Katarzyna Sokołowska*[1]*

Fig. S1.

Temperature and precipitation data in Wroclaw during 2016y (A) and between 2017-2020y (B).
The source of data was the Institute of Meteorology and Water Management (IMGW-PIB); website: https://danepubliczne.imgw.pl/

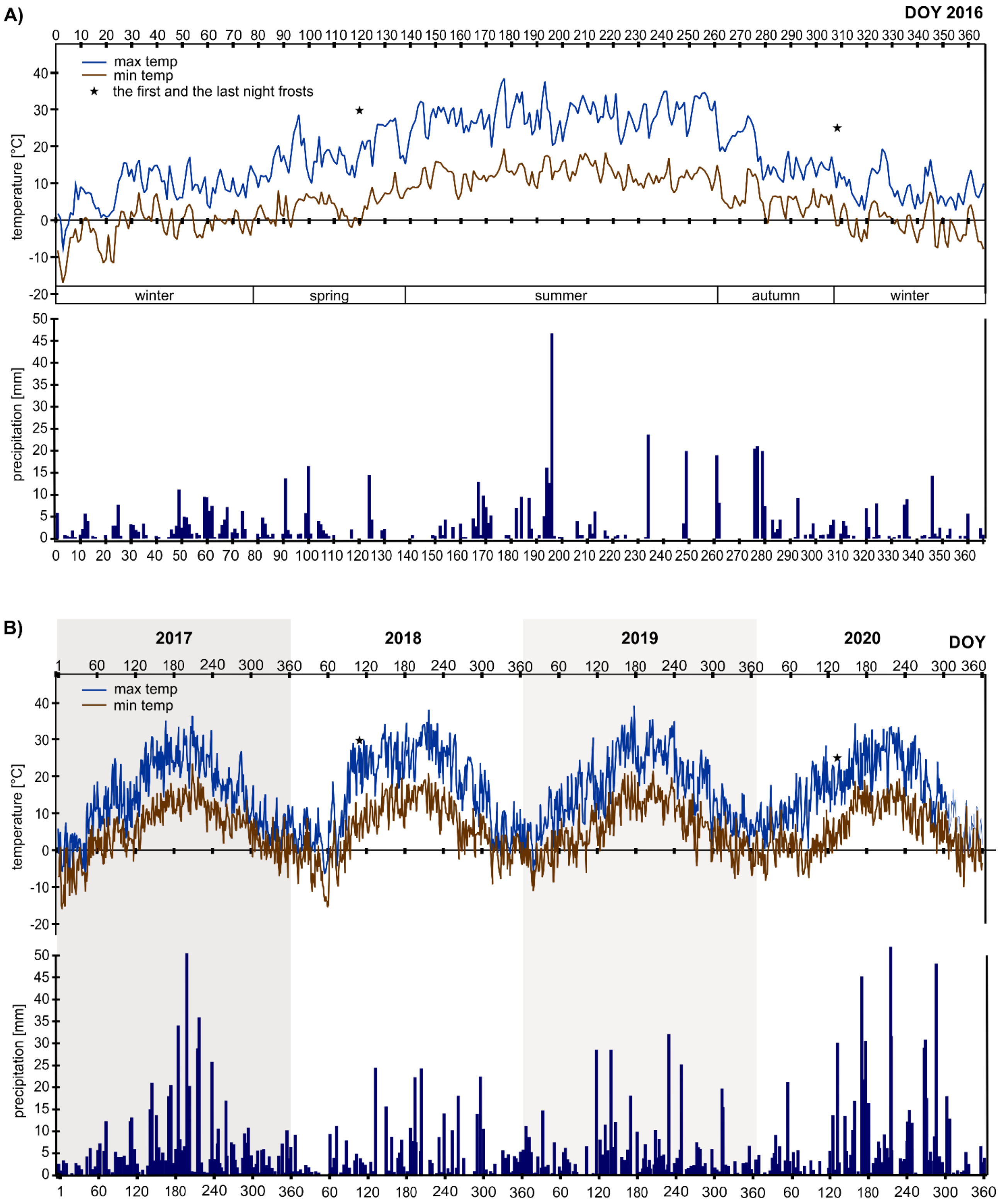
A)
DOY 2016
max temp
min temp
the first and the last night frosts
temperature [°C]
winter
spring
summer
autumn
winter
precipitation [mm]
B)
2017
2018
2019
2020
DOY
max temp
min temp
temperature [°C]
precipitation [mm]

Fig. S2

Anatomy of ash, maple and poplar wood, corresponding to Fig. 1. Structure of ash, maple, and poplar wood viewed on tangential (left) and radial (right) sections. Uni- and biseriate xylem rays, elongated axial parenchyma cells and fibers characterized by the presence of thick cell walls are shown in all species. All sections were viewed under bright-field microscopy. Abbreviations: ap, axial parenchyma; f, fibers; rp, ray parenchyma. Bars: 20 µm.

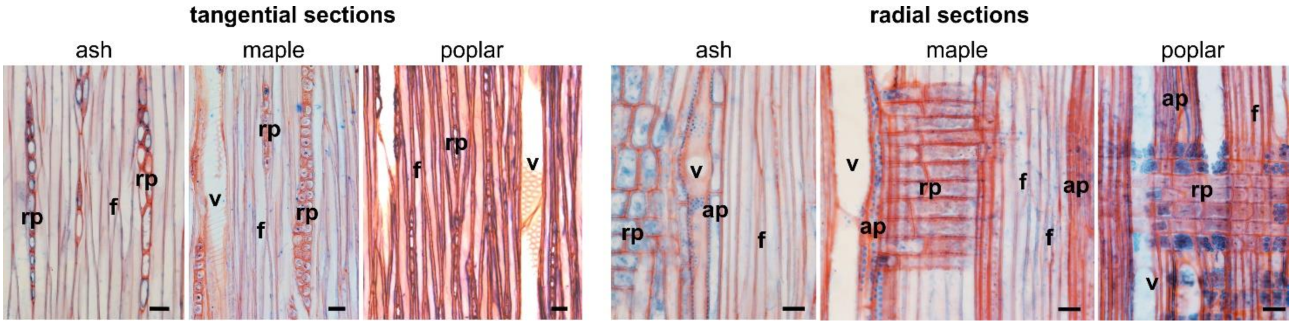

Fig. S3

Anatomical studies of ash and maple wood, corresponding to Fig. 2 (A) Intra-annual changes in cambial activity and xylem element formation during winter, spring, summer, and autumn in ash and maple trees; width of cambial region and the consecutive stages of xylem cell differentiation are marked with brackets; inlets represent magnification of cambial region and earlywood vessel differentiated from overwintering derivative; axial arrows on left side of images mark the width of annual growth ring. (B) Local activity of cambial meristem in ash stem, newly formed earlywood vessels are marked by yellow arrows. (C) Visualization of starch grains (white arrowheads) accumulated in XPC by staining the transverse hand-sections of maple and ash stems with 1% aqueous solution of iodine potassium iodide (IKI). All sections were viewed under bright-field microscopy. Abbreviations: ap, axial parenchyma; c, cambium; ev, earlywood vessel; msx, mature secondary xylem; lv, latewood vessel; pcg, post-cambial growth; rp, ray parenchyma; SCW+lig, secondary cell wall and lignification; sph, secondary phloem; sx, secondary xylem; v, vessel; VAC, vessel-associated cell. Bars: 20 µm.

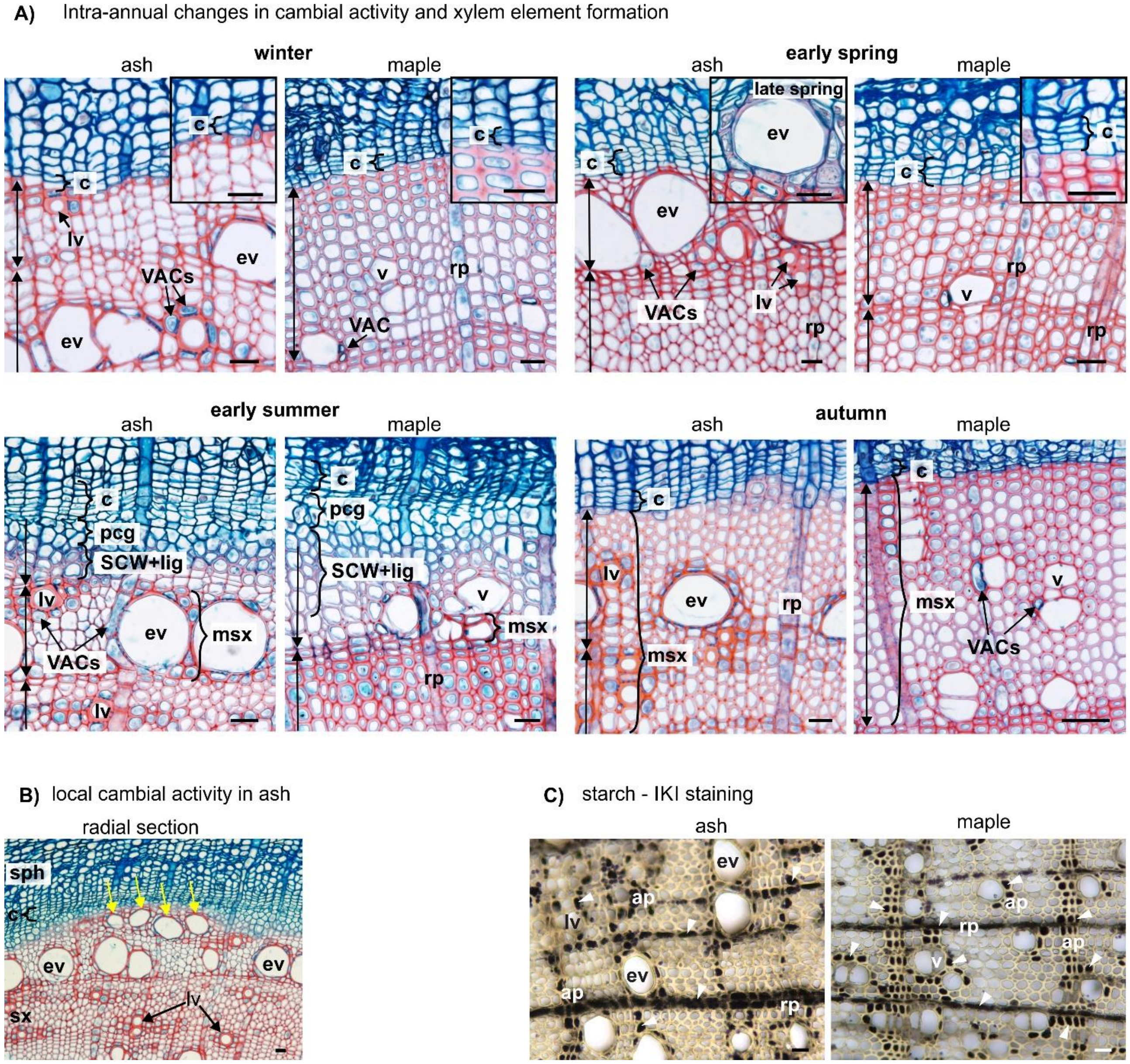

Fig. S4.

Multiple change point analysis in mean of seasonal changes in starch and soluble sugars, and in FM 4-64FX abundance of ash and maple trees, corresponding to Fig. 2B, D.

Multiple change point analysis of (A) seasonal changes in starch (upper row) and soluble sugars (lower row) amounts, based on data presented in Fig. 2B, and of (B) FM 4-64FX abundance in XPC (VAC and nonVAC), based on the data presented in Fig 2D. All figures, representing 50 randomly selected probes, present the posterior distribution estimated using the Markov Chain Monte Carlo method. Change-points were represented by peaks of the blue solid line. The narrower change-points are, the more certainty there is about the time at which the change-point occurred. Different change points' locations are associated with different regression models, as illustrated by the grey lines. The closer the grey lines are to each other, the more is the confidence about their estimation. Black dots represent the mean values of (A) starch or soluble sugar amounts or (B) FM 4-64FX abundance, green line marks the period of cambial activity. One change point for starch, two change points for soluble sugars, and two change points for seasonal changes in the dye abundance for ash and maple trees were detected.

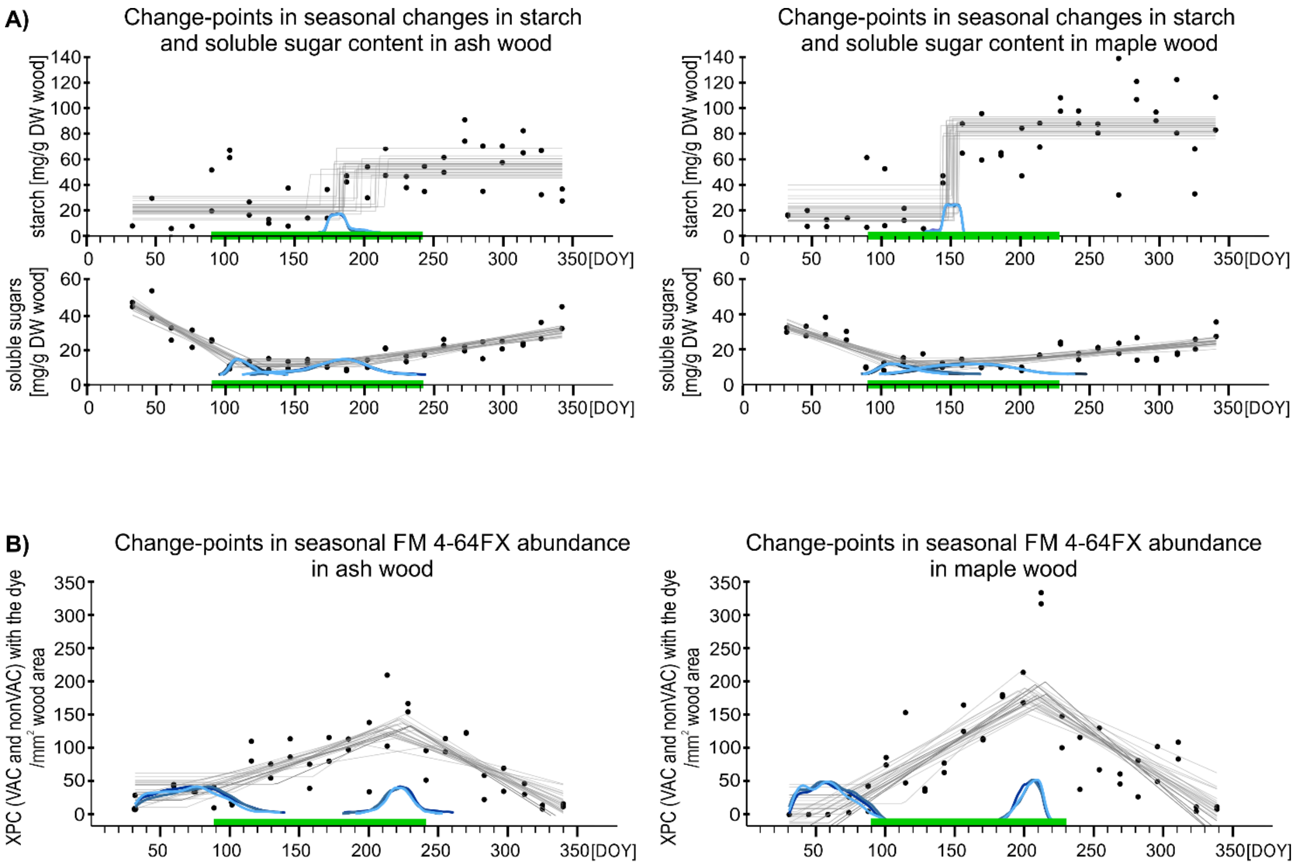

Fig. S5.

Annual variations in the content of soluble sugars and their compounds in ash and maple wood, corresponding to Fig. 2C. Relative abundance of particular sugars and sugar alcohols identified in wood samples collected during winter (46 and 341 DOY), spring (130 DOY), summer (201 DOY), and autumn (284 DOY) of 2016. All graphs represent the mean values (± SE); n=6; significant differences were indicated with stars. Kruskal-Wallis test was used to analyze the data, α = 0.05.

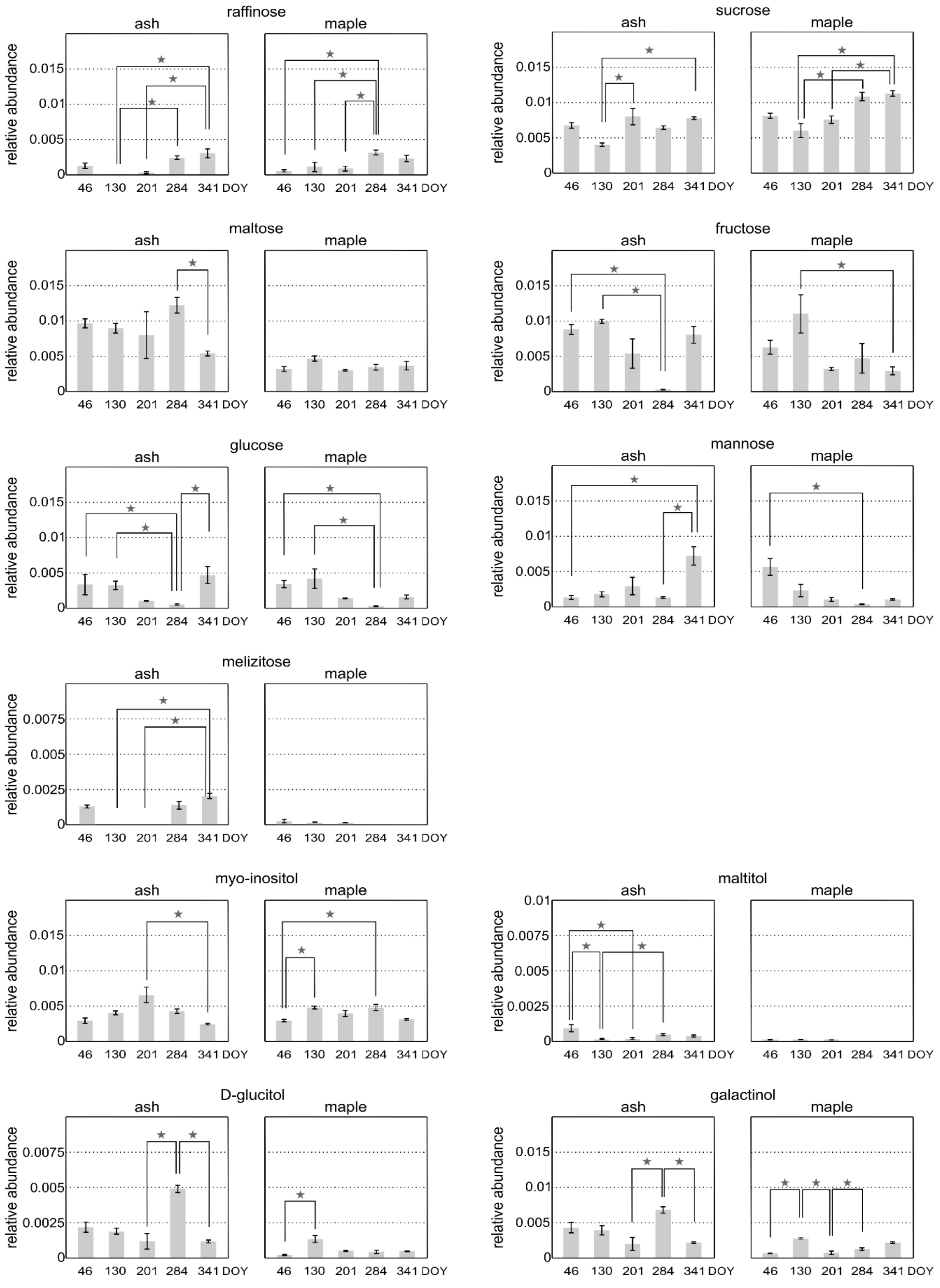

Fig. S6.

Seasonal differences in the FM 4-64FX localization in ash (A) and maple (B) wood during the four seasons, corresponding to Figures 2D, 3A. Distribution of FM 4-64FX dye in xylem parenchyma cells, specifically in VAC (white arrowheads) and nonVAC (empty arrowheads), on representative ash and maple wood profiles from winter, spring, summer, and autumn. Notably, VAC with internalized FM 4-64FX dye belong both to the axial and ray parenchyma systems. Abbreviations: ap, axial parenchyma; rp, ray parenchyma. The lumens of the selected vessel elements are outlined, dashed lines mark the shapes of the analyzed wood profiles. Bars: 50 µm.

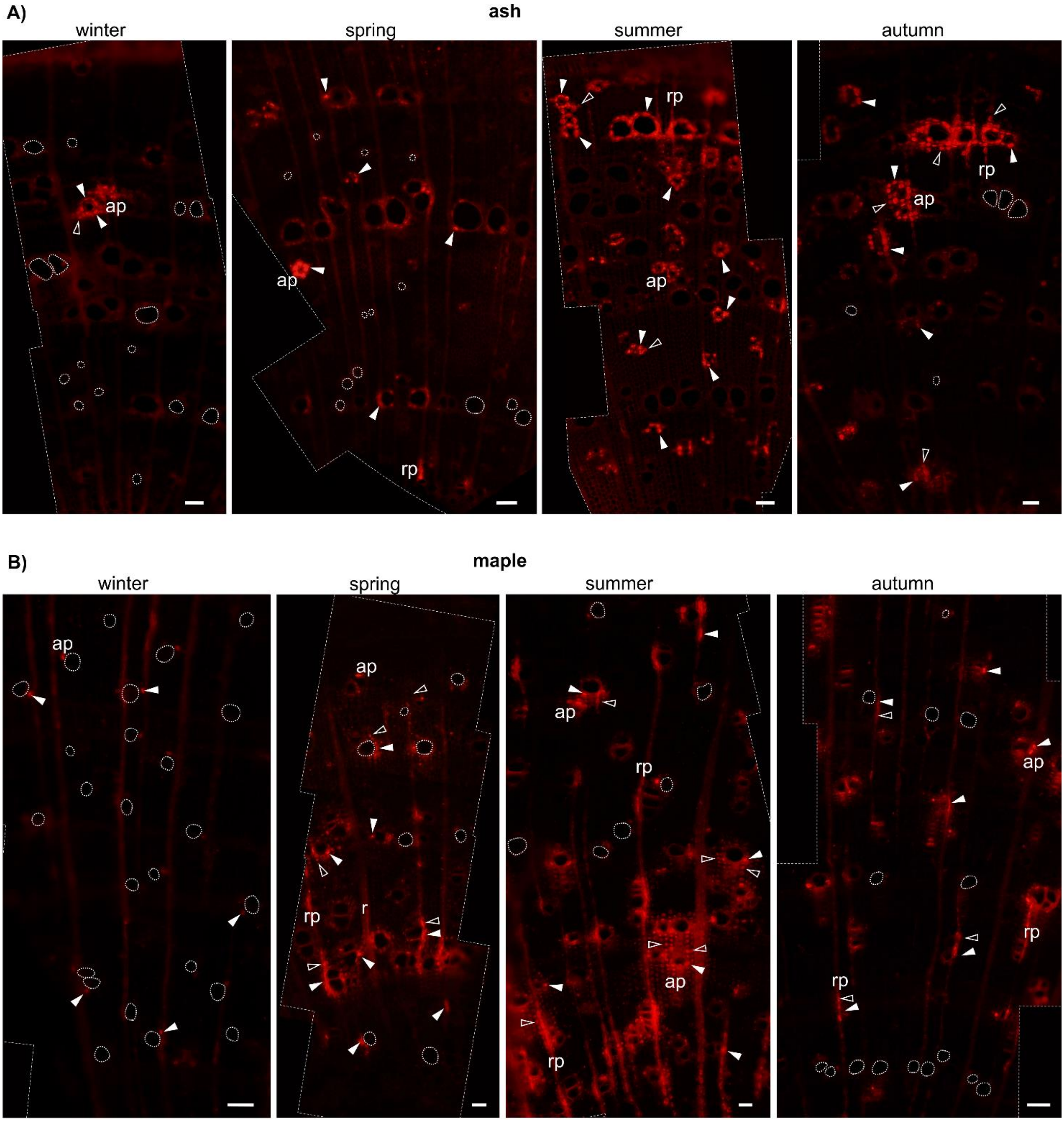

Fig. S7.

Seasonal differences in the FM 4-64FX localization in poplar wood during the four seasons, corresponding to Figure 3A. Distribution of FM 4-64FX dye in xylem parenchyma cells, specifically in VAC (white arrowheads) and nonVAC (empty arrowheads), on representative poplar wood profiles from winter, spring, summer, and autumn seasons. Notably, VAC with internalized FM 4-64FX dye belong mostly to the ray parenchyma systems. Abbreviations: ap, axial parenchyma; rp, ray parenchyma. The lumens of the selected vessel elements are outlined. Bars: 50 µm.

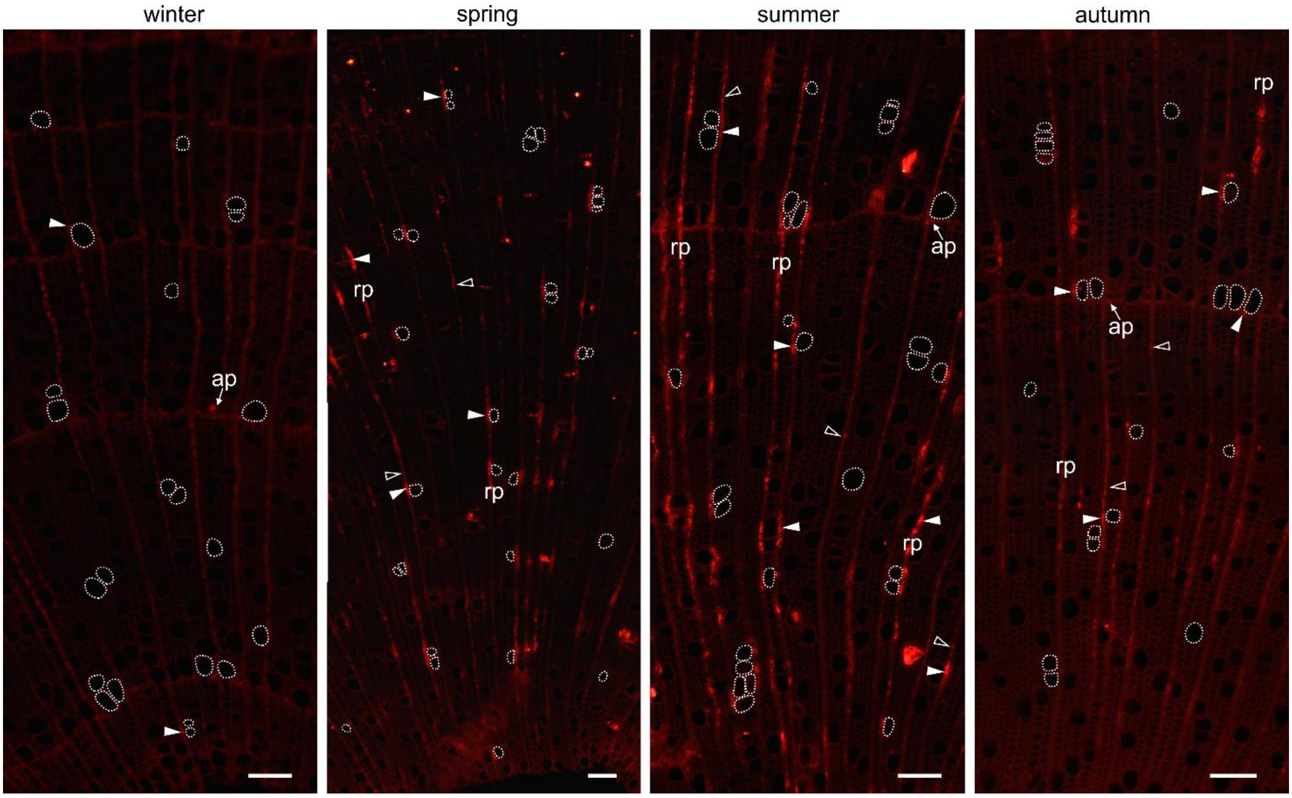

Fig. S8.

Changes in expression levels of sucrose transporters (*PttSUT3*, *PttSUT4*), clathrin-related genes (*PttCHC*, *PttCLC*), and remorins (*PttREM1.4*, *PttREM6.1*); results based on *EF1b* reference gene corresponding to Figure 3. Results from the same samples that were presented in Fig. 3, but based on a different reference gene, i. e. *EF1b*. significant differences are marked with different letters: *PttSUT4* with p=0.008017, *PttCLC* with p=0.00988, *PttREM1.4* with p=0.036570; Kruskal-Wallis test, α = 0.05. All graphs represent mean values (± SE).

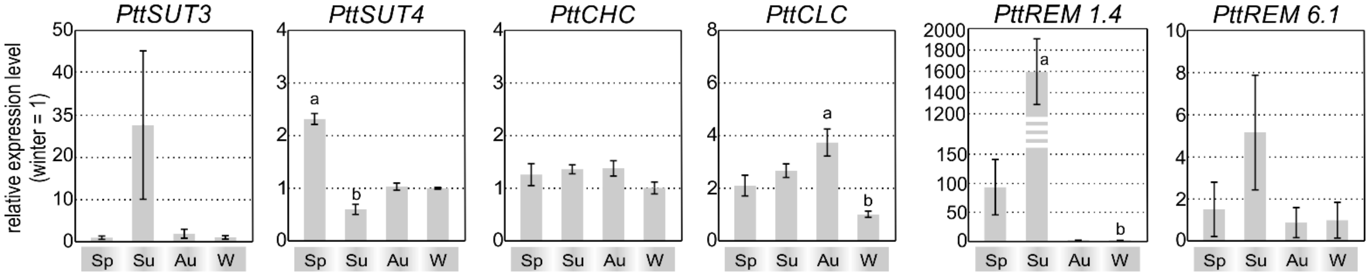

Fig. S9.

Localization of FM dyes and callose in the xylem parenchyma cells of ash and poplar wood, corresponding to Figures 4 and 5. (A) The fluorescent signal of FM 1-43FX localized to both types of xylem parenchyma cells – VAC and nonVAC - in ash (left) and poplar (right) wood, white arrowheads indicate the dye presence in the simple pits connecting neighbouring xylem parenchyma cells. (B) Localization of FM 4-64FX dye (red) and anti-callose (anti-1,3 β-glucan) antibody (blue) in ash (left) and poplar (right) wood. The fluorescent signals of FM 4-64FX are visible in both VAC and nonVAC and in simple pits connecting these cells, the signals corresponding to anti-callose antibody are marked by white arrowheads. Abbreviations: nonVAC, non-vessel-associated cell; v, vessel; VAC, vessel-associated cell; the lumens of the selected vessel elements are outlined. Bars: 10 µm.

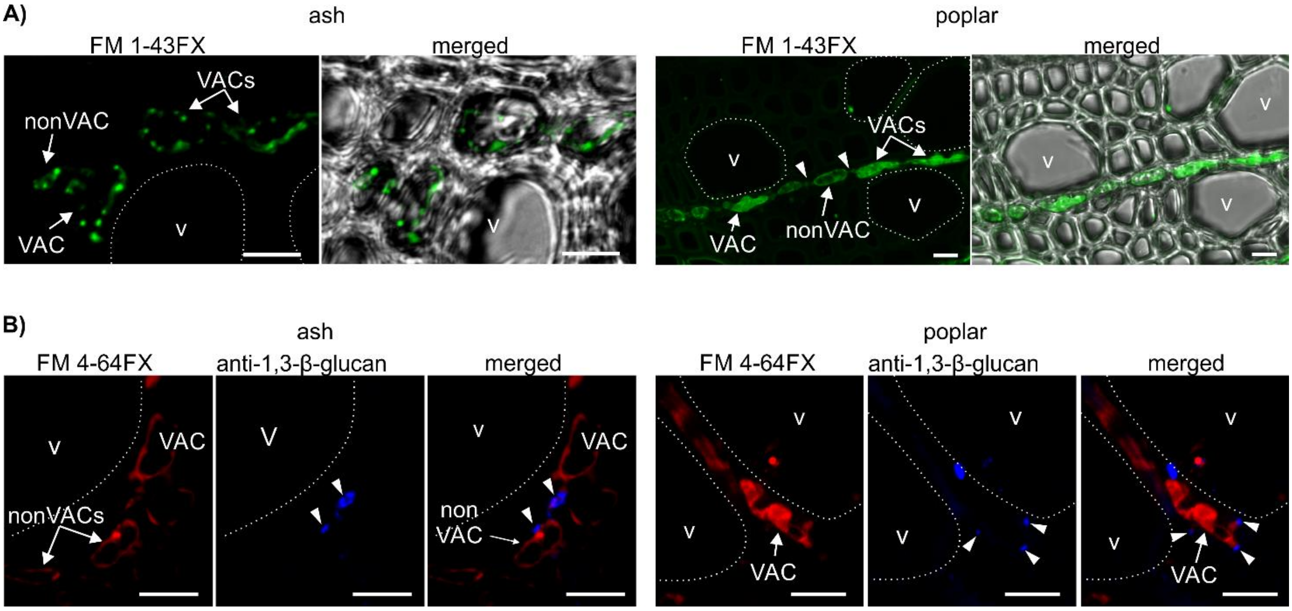

Table S1.
Sequences of oligonucleotides used for qRT-PCR, corresponding to Figure 3B.

Gene ID	Forward Primer	Reverse Primer
PttCHC potri.008g070600	ATGGAGACAGCATCACAGTCTGG	AGGCAAGCTCAAGGGCAATGTC
PttCLC potri.004g040100	AGGATGTCAAAGATCCCGTAACCG	TTTGCTCCTGACGTGGTTGCAC
PttREM1.4 potri.001g107000	GTTGCAGATCCACCTGCTACTG	GCACAGCATCTCTGTCATTTGGC
PttREM6.1 potri.008g144300	GCAACAACACCAATTCGCAGTCC	TTGGAGATGAAGCTGGTGCGGTTC
PttSUT3 potri.019g085800	TGGTGTCTGTAGCAAGTGGACCTT	GGAATGCAGCAGTGACAGTCCTT
PttSUT4 potri.002g106900	ATCCTTGGGACTTGGACAAGGGTT	TGATCGAGGAATACCCAAGATGGC
PttUBCE2a potri.019g039200	AGTACAACCGCAGAGTTCGTGAAG	ATGGACTATCAGTCAGCCGTCCAG
PttEF1b potri. 001G224700	TGAGGATCTCTGGTGTCGAAG	GTCTCAGCAGATGGAGGAGTG

Dataset S1

The R code used to analyse the change points in intra-annual changes in NSC content or FM 4-64FX dye abundance in ash and maple wood.

```
library("tidyverse")

library("readxl")

library("ggpubr")

library("mcp") # need to install https://sourceforge.net/projects/mcmc-jags/

theme_set(theme_pubr(base_size = 12)) # figures


## Reading data


maple_ash <- read_excel("data/baza.xlsx")

temp <- read_excel("data/baza_temp.xlsx")


## Data preparation


maple_ash <- maple_ash %>%

  mutate(across(where(is.character), as.factor)) %>%

  mutate(Species = fct_relevel(Species, "maple"))


maple_ash_means <- maple_ash %>%

  select(-c(Subject, `Cambial activity`, `Postcambial growth`,

    `SCW + lignification`)) %>%

  pivot_longer(Starch_1:`Tracer transport_9`, names_to = c(".value", "Repeats"),
```

```
    names_sep = "_") %>%
  group_by(DOY, Species) %>%
  summarise(
    Starch = mean(Starch, na.rm = TRUE),
    Sugars = mean(`Soluble sugars`, na.rm = TRUE),
    Transport = mean(`Tracer transport`, na.rm = TRUE)
  ) %>%
  ungroup()

maple_ash_means <- maple_ash %>%
  filter(Subject %in% c("A1", "F1")) %>%
  select(DOY:`SCW + lignification`) %>%
  select(-Subject) %>%
  left_join(maple_ash_means)

cambial <- maple_ash_means %>%
  filter(`Cambial activity` == "Y") %>%
  mutate(`Cambial activity` = -1)

pc_growth <- maple_ash_means %>%
  filter(`Postcambial growth` == "Y") %>%
  mutate(`Postcambial growth` = -0.6)

scw_lig <- maple_ash_means %>%
  filter(`SCW + lignification` == "Y") %>%
  mutate(`SCW + lignification` = -0.2)

maple_ash <- maple_ash %>%
  pivot_longer(c(Starch_1:`Tracer transport_9`), names_to = c(".value","Sample"),
    names_sep = "_") %>%
  group_by(DOY, Species, Subject) %>%
  summarise(
    Starch = mean(Starch, na.rm = TRUE),
    Sugars = mean(`Soluble sugars`, na.rm = TRUE),
```

```
    Transport = mean(`Tracer transport`, na.rm = TRUE)
  ) %>%
  ungroup()

maple <- maple_ash %>%
  filter(Species == "maple") %>%
  select(-Species)

ash <- maple_ash %>%
  filter(Species == "ash") %>%
  select(-Species)

cambial_maple <- maple_ash_means %>%
  filter(`Cambial activity` == "Y", Species == "maple")

cambial_ash <- maple_ash_means %>%
  filter(`Cambial activity` == "Y", Species == "ash")

## Fitting models

fit_maple_starch <- mcp(list(Starch ~ 1, ~ 1), drop_na(maple, Starch), par_x = "DOY")
fit_maple_sugars <- mcp(list(Sugars ~ DOY, ~ 0, ~ 0 + DOY), drop_na(maple, Sugars),
  par_x = "DOY")
fit_maple_transport <- mcp(list(Transport ~ 1, ~ 0 + DOY, ~ 0 + DOY),
  drop_na(maple, Transport), par_x = "DOY")

fit_ash_starch <- mcp(list(Starch ~ 1, ~ 1), drop_na(ash, Starch), par_x = "DOY")
fit_ash_sugars <- mcp(list(Sugars ~ DOY, ~ 0, ~ 0 + DOY), drop_na(ash, Sugars),
  par_x = "DOY")
fit_ash_transport <- mcp(list(Transport ~ 1, ~ 0 + DOY, ~ 0 + DOY),
  drop_na(ash, Transport), par_x = "DOY")

## Results
```

```
summary(fit_maple_starch)
summary(fit_maple_sugars)
summary(fit_maple_transport)
summary(fit_ash_starch)
summary(fit_ash_sugars)
summary(fit_ash_transport)

## Figures

my_breaks <- unique(maple$DOY)
my_breaks <- my_breaks[seq(1, length(my_breaks), by = 2)]

plot(fit_maple_starch) +
  geom_line(aes(DOY, `Cambial activity`),
    mutate(cambial_maple, `Cambial activity` = 0),
    linewidth = 1.5, col = "green3") +
  scale_x_continuous(breaks = my_breaks)

plot(fit_maple_sugars) +
  geom_line(aes(DOY, `Cambial activity`),
    mutate(cambial_maple, `Cambial activity` = 0.65),
    linewidth = 1.5, col = "green3") +
  labs(y = "Soluble sugars") +
  scale_x_continuous(breaks = my_breaks)

plot(fit_maple_transport) +
  geom_line(aes(DOY, `Cambial activity`),
    mutate(cambial_maple, `Cambial activity` = -12),
    linewidth = 1.5, col = "green3") +
  labs(y = "Tracer transport") +
  scale_x_continuous(breaks = my_breaks)

plot(fit_ash_starch) +
  geom_line(aes(DOY, `Cambial activity`),
```

```
    mutate(cambial_ash, `Cambial activity` = 0.15),
    linewidth = 1.5, col = "green3") +
  scale_x_continuous(breaks = my_breaks)

plot(fit_ash_sugars) +
  geom_line(aes(DOY, `Cambial activity`),
    mutate(cambial_ash, `Cambial activity` = 0.67),
    linewidth = 1.5, col = "green3") +
  labs(y = "Soluble sugars") +
  scale_x_continuous(breaks = my_breaks)

plot(fit_ash_transport) +
  geom_line(aes(DOY, `Cambial activity`),
    mutate(cambial_ash, `Cambial activity` = 0),
    linewidth = 1.5, col = "green3") +
  labs(y = "Tracer transport") +
  scale_x_continuous(breaks = my_breaks)
```

Video S1.
Fluorescence Recovery After Photobleaching (FRAP) in *Fraxinus excelsior* (ash). Testing intercellular transport of FM 4-64FX between living xylem parenchyma cells.

Video S2.
Fluorescence Recovery After Photobleaching (FRAP) in *Populus tremula* x *tremuloides* (poplar). Testing intercellular transport of FM 4-64FX between living xylem parenchyma cells.
